# Long-term exposure to particulate matter on cardiovascular and respiratory diseases in low- and middle-income countries: A systematic review and meta-analysis

**DOI:** 10.3389/fpubh.2023.1134341

**Published:** 2023-03-28

**Authors:** Juanmei Guo, Guorong Chai, Xuping Song, Xu Hui, Zhihong Li, Xiaowen Feng, Kehu Yang

**Affiliations:** ^1^School of Management, Lanzhou University, Lanzhou, China; ^2^Evidence-based Social Sciences Research Center, School of Public Health, Lanzhou University, Lanzhou, China; ^3^Key Laboratory of Evidence-Based Medicine and Knowledge Translation of Gansu Province, Lanzhou, China

**Keywords:** particulate matter, cardiovascular diseases, respiratory diseases, low- and middle-income countries, long-term exposure

## Abstract

**Background:**

Long-term exposure to particulate matter (PM) has essential and profound effects on human health, but most current studies focus on high-income countries. Evidence of the correlations between PM and health effects in low- and middle-income countries (LMICs), especially the risk factor PM_1_ (particles < 1 μm in size), remains unclear.

**Objective:**

To explore the effects of long-term exposure to particulate matter on the morbidity and mortality of cardiovascular and respiratory diseases in LMICs.

**Methods:**

A systematic search was conducted in the PubMed, Web of Science, and Embase databases from inception to May 1, 2022. Cohort studies and case-control studies that examine the effects of PM_1_, PM_2.5_, and PM_10_ on the morbidity and mortality of cardiovascular and respiratory diseases in LMICs were included. Two reviewers independently selected the studies, extracted the data, and assessed the risk of bias. Outcomes were analyzed *via* a random effects model and are reported as the relative risk (RR) with 95% CI.

**Results:**

Of the 1,978 studies that were identified, 38 met all the eligibility criteria. The studies indicated that long-term exposure to PM_2.5_, PM_10_, and PM_1_ was associated with cardiovascular and respiratory diseases: (1) Long-term exposure to PM_2.5_ was associated with an increased risk of cardiovascular morbidity (RR per 1.11 μg/m^3^, 95% CI: 1.05, 1.17) and mortality (RR per 1.10 μg/m^3^, 95% CI: 1.06, 1.14) and was significantly associated with respiratory mortality (RR 1.31, 95% CI: 1.25, 1.38) and morbidity (RR 1.08, 95% CI: 1.02, 1.04); (2) An increased risk of respiratory mortality was observed in the elderly (65+ years) (RR 1.21, 95% CI: 1.00, 1.47) with long-term exposure to PM_2.5_; (3) Long-term exposure to PM_10_ was associated with cardiovascular morbidity (RR 1.07, 95% CI 1.01, 1.13), respiratory morbidity (RR 1.43, 95% CI: 1.21, 1.69) and respiratory mortality (RR 1.28, 95% CI 1.10, 1.49); (4) A significant association between long-term exposure to PM_1_ and cardiovascular disease was also observed.

**Conclusions:**

Long-term exposure to PM_2.5_, PM_10_ and PM_1_ was all related to cardiovascular and respiratory disease events. PM_2.5_ had a greater effect than PM_10_, especially on respiratory diseases, and the risk of respiratory mortality was significantly higher for LMICs than high-income countries. More studies are needed to confirm the effect of PM_1_ on cardiovascular and respiratory diseases.

## Introduction

Air pollution has long been recognized as both a public health problem and a social problem, and air pollutants are classified as carcinogens by the International Agency for Research on Cancer (IARC) (1). According to the latest urban air quality database information from the World Health Organization (WHO), 56 percent of cities in high-income countries with a population over 100,000 do not comply with WHO air quality guidelines, but in low- and middle-income countries (LMICs), the figure is 98%. In the past few years, air pollution has become increasingly serious. The public health significance of PM pollution is much greater than that of other air pollutants. PM pollution is associated with haze, and of all the air pollutants, it is most closely connected to adverse health effects (2). Epidemiological research has suggested that particulate air pollution is associated with many adverse health outcomes, including increased mortality and morbidity caused by lung and heart diseases (3). In the 2005 revision of the Air Quality Guidelines (AQG), the WHO defined PM as a major global air pollutant. PM pollution can result in multi-system damage, especially to the respiratory and cardiovascular systems. The evidence of the respiratory and cardiovascular disease effects of respirable PM with aerodynamic diameters below 2.5 and 10 mm (i.e., PM_2.5_, and PM_10_) is growing (4).

Several studies have reported a global correlation between PM and respiratory and cardiovascular diseases (5–7). In fact, short-term exposure to PM_10_ and PM_2.5_ has been associated with respiratory and cardiovascular mortality, as well as daily all-cause mortality, in over 600 cities (8). Current research has concentrated on the acute health effects of PM pollutants. However, long-term effects remain a significant issue, particularly for decision-making regarding better air pollution control and assessing the long-term effects on public health (9).

The WHO estimates that air pollution results in over approximately one million premature deaths throughout the world each year (10). According to a recent report on the global burden of disease, particulate air pollution leads to 3.1 million deaths worldwide each year, and 22% of disability-adjusted life years (DALYs) are caused by cardiovascular disease (11).

LMICs have poor health care capabilities, but they bear a high proportion of the global morbidity and mortality caused by air pollution. Increased exposure to risk factors throughout life (e.g., particulate pollution and smoking) is associated with higher cardiovascular and respiratory disease prevalence in LMICs, but the lack of treatment availability increases the avoidable harm. Numerous current studies have shown the effects of PM_2.5_ and PM_10_ in high-income countries; however, less attention is paid to LMICs, particularly the effects of PM_1_. This study aims to comprehensively review existing efforts in order to facilitate future studies.

## Methods

A PRISMA 2020 (Preferred Reporting Items for Systematic Reviews and Meta-Analyses) statement was utilized as a guide for reporting this systematic review (12, 13). We used data extracted from published articles; therefore, this study has no discernible ethical issues.

### Search strategy

Systematic reviews offer a unique advantage in decision-making in health care (12). PubMed, Embase and Web of Science databases were systematically searched utilizing following terms: (air pollution OR particulate matter) AND (respiratory^*^ OR cardiovascular^*^) AND (morbidit^*^ OR hospitalization^*^ OR hospitalization^*^ OR death^*^ OR mortalit^*^ OR outpatien^*^) AND (case-control OR cohort) AND (developing country). We restricted the search from inception to May 1, 2022, and no limitations were placed on the publication dates. Furthermore, we manually searched the lists of references contained in the studies to determine additional relevant studies. The details for each database's search strategy can be found in the online Supplementary material. All manuscripts were uploaded to Rayyan and screened independently by two reviewers (XF and ZL). Any disagreements were resolved through discussion and consultation with a third member (XH) of the review team until a consensus was reached.

### Selection of studies

The titles and abstracts of all independently acquired articles were reviewed by two of the study authors (XF and ZL), and the relevant studies were then identified through full-text assessment. The reasons for exclusion during the full-text screening were recorded. Any disagreements that arose were resolved through discussion and, if necessary, with the involvement of the third author. Case-control or cohort studies assessing the effects of PM_10_, PM_2.5_ and PM_1_ on the morbidity and mortality of cardiovascular and respiratory diseases in LMICs were enrolled. The studies were included if the following criteria were met: (1) the type of study was limited to cohort and case-control studies; (2) studies in which PM_1_, PM_2.5_ and PM_10_ were included as pollutants and studies reporting long-term exposure (months to years) to ambient air PM_1_, PM_2.5_, and PM_10_ expressed as a concentration unit (μg/m^3^) were included; (3) the study locations were low- and middle-income countries (LMICs); (4) the studies were conducted according to the International Classification of Diseases (ICD), 9th or 10th Revision, and included cardiovascular disease (ICD-9 codes 390-459, ICD-10 codes I00-I99) or respiratory disease (ICD-9 codes 460-519, ICD-10 codes J00-J99); (5) the studies included morbidity or mortality as an outcome; (6) estimates were expressed as the relative risk (RR), OR or HR with 95% CI, or sufficient information was included for calculation; (7) the publication language was limited to English.

Articles were excluded according to the following: (1) studies reporting occupational exposure (measured in the workplace) or exclusively indoor exposure to PM_1_, PM_2.5_ and PM_10_ were excluded; (2) studies evaluating disease progression in patients suffering from respiratory or cardiovascular diseases [for instance, asthma or chronic obstructive pulmonary disease (COPD)] and exposed to pollutants; (3) studies linked to seasonality; (4) duplicate studies, commentaries, summaries, editorials, letters, and conference abstracts; (5) the information provided in the results was insufficient for data extraction.

### Extraction of data

XF and ZL independently extracted the indicated data from the included cohort and case-control studies. If disputes remained after discussion, a third investigator (XH) was engaged to resolve the conflict. The following data were extracted from all included studies and entered into a Microsoft Excel database (Version 2014 Microsoft, USA): author, location, year of publication, study design, study duration, study group, pollutant, type of disease, number of events, health outcomes, and specific risk estimates.

### Risk of bias assessment

Two reviewers (JG and XF) evaluated the underlying risk of bias independently for all the included studies with the Newcastle-Ottawa Quality Assessment Scale (NOS) (14); disagreements were discussed and resolved by consensus with the third review author (XH). The NOS provides scores of 0–9 according to selection, comparability, and outcome evaluation. Studies with scores of 0–3, 4–6, and 7–9 were respectively considered to be low-, medium-, and high-quality studies.

### Statistical methods

For meta-analysis, RR was used as an effect estimate, and OR for case crossover studies and HR for cohort studies were considered equivalent to RR (15, 16). Where multiple estimates existed in the primary study, maximum adjusted model estimates were extracted to minimize the risk of underlying unmeasured confounding. RR for morbidity and mortality was used as impact values and was converted to a standardized increment (10 μg/m^3^) for PM concentration. The following formula was used to calculate the standardized risk estimates:


(1) $${\text{RR}}_{\left(\text{standardised}\right)}=\text{RR}{\;}_{\left(\text{original}\right)}^{\text{Increment}(10)/\text{Increment}(\text{original})}$$

For this meta-analysis, a random-effects model was constructed to anticipate significant heterogeneity among studies. We used the I^2^ statistic to estimate the degree of heterogeneity for each analysis. Values of I^2^ < 25%, 25–50%, and >50% respectively represent low, moderate, and high heterogeneity. If we identified substantial unexplained heterogeneity, we reported it and explored potential influencing factors with a prespecified subgroup analysis of the results of each data-sufficient synthesis, including sex (male vs. female), age (<65 years vs. >65 years), type of study (cohort vs. case-control), and type of disease. If a study reported subgroup data separately, we directly used the corresponding data for our analysis. Publication bias was assessed by Egger's regression test when the outcome included more than 10 studies.

All analyses were carried out with Stata software (Version 15.0, Stata Corp., College Station, TX, USA), and statistical significance was deemed to be two-sided *P* < 0.05.

## Results

**Figure 1:**
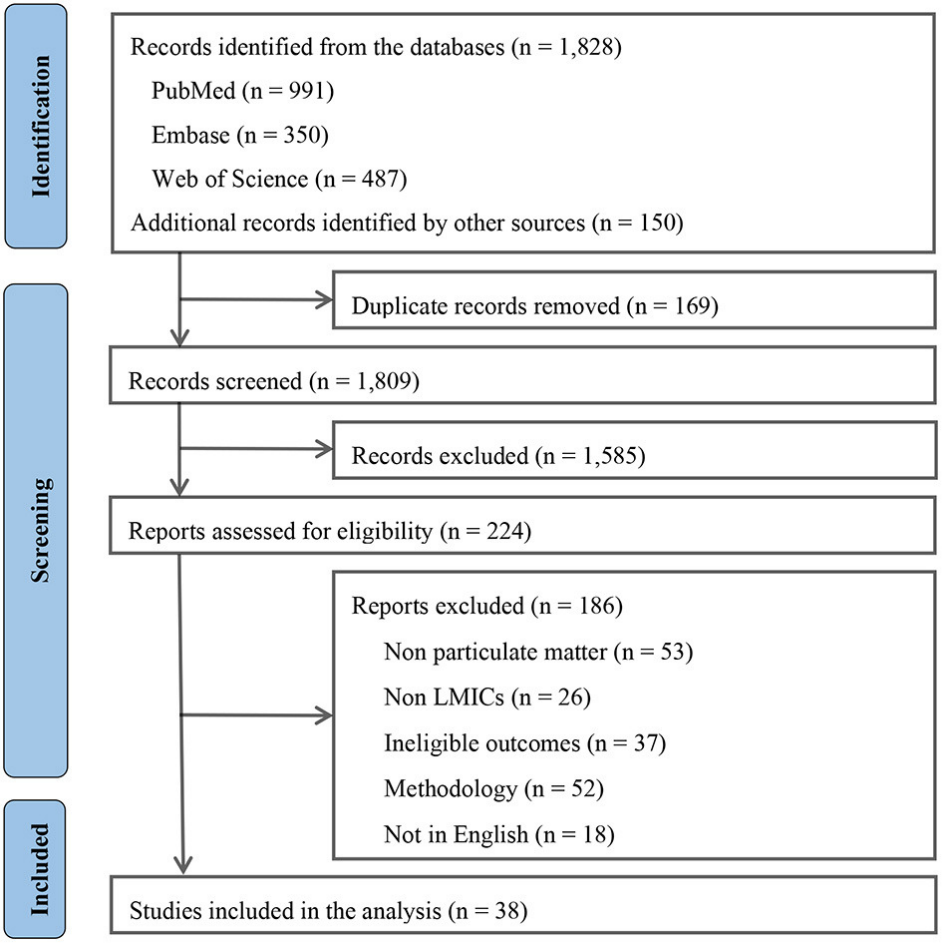
Flow chart for study inclusion.

Our search yielded 1,978 unique records, of which 224 were potentially eligible and subjected to further full-text review. Ultimately, 38 studies (6, 7, 17–51) of long-term PM exposure met the criteria and were chosen for meta-analysis (Figure 1).

Table 1 presents the main characteristics of the included studies. A number of studies assessed the morbidity and mortality effects of long-term exposure to PM_2.5_ (*n* = 24) (22, 23, 25, 26, 28, 30, 33–42, 44–50, 52) or PM_10_ (*n* = 19) (6, 7, 17–21, 24, 27–29, 31, 32, 34, 37, 38, 43, 50, 51), but there were few studies of PM_1_ (*n* = 2) (37, 40). The vast majority utilized a cohort study design (*n* = 35) (6, 7, 17–20, 22–33, 35, 36, 38–51), and only three articles (21, 34, 37) used a case-control study design. Three studies (7, 30, 50) analyzed both cardiovascular and respiratory diseases; 22 studies (17, 18, 20–22, 28, 31, 33–38, 40–42, 45–47, 49, 51) investigated only cardiovascular diseases, and 13 studies (6, 19, 23–27, 29, 32, 39, 43, 44, 48) assessed respiratory diseases. Seventeen (20, 21, 23, 24, 27, 28, 31, 32, 34–36, 38, 41, 43, 47, 48, 51) of the 38 studies reported morbidity as an outcome variable; 17 studies (6, 7, 17–20, 22, 25, 26, 29, 30, 33, 37, 39, 40, 46, 49) reported mortality, and 4 studies (42, 44, 45, 50) reported both morbidity and mortality. Thirty-five studies were conducted in China (6, 7, 17–41, 44–50); 2 studies were performed in Thailand (43, 51), and the remaining study (42) used data from 21 different countries.

**Table 1:** Features of the included studies regarding long-term PM exposure.

**References**	**Area**	**Species**	**Mean/median exposure (μg/m^3^)**	**Study period**	**Study design**	**Sample size**	**Age (years)**	**Disease(s)**	**Outcome(s)**	**NOS score**
Zhang et al. (17)	Northern China	PM_10_	154.0	1998–2009	Cohort study	12,584	35–103	CVD	Mortality	7
Dong et al. (19)	Shenyang, China	PM_10_	154.0	1998–2009	Cohort study	9,941	≥25	RSD	Mortality	8
Zhang et al. (18)	Northern China	PM_10_	144.0	1998–2009	Cohort study	39,054	≥40	CVD	Mortality	7
Zhou et al. (7)	25 cities in China	PM_10_	104.0	1990–1991	Cohort study	71,431	≥40	CVD and RSD	Mortality	5
Tseng et al. (52)	Taiwan, China	PM_2.5_	30.5	1989–2008	Cohort study	43,227	≥26	CVD	Mortality	7
Hwang et al. (21)	Taiwan, China	PM_10_	60.3	2001–2007	Case-control study	1,087	Infants	CVD	Morbidity	6
Yin et al. (22)	44 areas in China	PM_2.5_	47.3	2000–2005	Cohort study	186,399	40–79	CVD	Mortality	5
Lai et al. (23)	Taiwan, China	PM_2.5_	27.8	2005–2012	Cohort study	106,678	≥18	RSD	Morbidity	5
Jin et al. (20)	Lanzhou, China	PM_10_	143.8	2010–2012	Cohort study	8,969	Infants	CVD	Morbidity	7
Liu et al. (24)	Shanghai, China	PM_10_	82.0	2011–2012	Cohort study	3,358	4–6	RSD	Morbidity	5
Peng et al. (25)	Shanghai, China	PM_2.5_	53.5	2003–2013	Cohort study	4,444	≥14	RSD	Mortality	6
Chen et al. (6)	Northern China	PM_10_	44.3	1998–2009	Cohort study	39,054	Mean 44.29	RSD	Mortality	7
Wong et al. (26)	Hongkong, China	PM_2.5_	33.7	1998–2001	Cohort study	66,820	≥65	RSD	Mortality	5
Deng et al. (27)	Changsha, China	PM_10_	90.0	2011–2012	Cohort study	2,598	3–6	RSD	Morbidity	7
Zhang et al. (28)	Wuhan, China	PM_2.5_, PM_10_	65.6/101.7	2012–2013	Cohort study	105,988	Infants	CVD	Morbidity	8
Chen et al. (29)	Four cities in China	PM_10_	144.3	1999–2009	Cohort study	39,054	≥42	RSD	Mortality	7
Yin et al. (30)	45 areas in China	PM_2.5_	43.7	1990–1991	Cohort study	189,793	≥40	CVD and RSD	Mortality	7
Ren et al. (31)	Beijing, China	PM_10_	104.1	2009–2012	Cohort study	30,669	Infants	CVD	Morbidity	5
Jiang et al. (32)	Changsha, China	PM_10_	110.0	2011–2012	Cohort study	2,598	3-6	RSD	Morbidity	6
Yang et al. (33)	Hongkong, China	PM_2.5_	42.2	1998–2011	Cohort study	61,386	≥65	CVD	Mortality	8
Huang et al. (34)	Taiwan, China	PM_2.5_, PM_10_	30.6/53.0	2007–2014	Case-control study	5,474	Infants	CVD	Morbidity	7
Huang et al. (35)	15 provinces in China	PM_2.5_	64.9	1992–2008	Cohort study	117,575	≥18	CVD	Morbidity	6
Huang et al. (36)	China	PM_2.5_	77.7	2014–2015	Cohort study	59,456	≥18	CVD	Morbidity	8
Chen et al. (37)	China	PM_1_, PM_2.5_, PM_10_	63.3/80.6/134.9	2007–2008	Case-control study	12,291	≥18	CVD	Mortality	6
Mao et al. (38)	Henan, China	PM_2.5_, PM_10_	72.8/131.5	2015–2017	Cohort study	39,259	18-79	CVD	Morbidity	7
Sun et al. (39)	Hongkong, China	PM_2.5_	35.3	1998–2001	Cohort study	58,643	≥65	RSD	Mortality	8
Yang et al. (40)	Northeastern China	PM_1_, PM_2.5_	65.9/82.0	2006–2008	Cohort study	24,845	18-74	CVD	Mortality	6
Bo et al. (41)	Hongkong, China	PM_2.5_	26.6	2001–2014	Cohort study	134,978	≥18	CVD	Morbidity	7
Hystad et al. (42)	Multiple countries	PM_2.5_	47.5	2003–2018	Cohort study	157,436	35-70	CVD	Morbidity, mortality	8
Ruchiraset et al. (43)	Thailand	PM_10_	74.0	2003–2014	Cohort study	41,085	≥18	RSD	Morbidity	5
Li et al. (44)	China	PM_2.5_	65.0	2000–2015	Cohort study	118,551	≥18	RSD	Morbidity, mortality	7
Liang et al. (45)	China	PM_2.5_	67.4	2000–2015	Cohort study	127,840	≥18	CVD	Morbidity, mortality	8
Yang et al. (46)	China	PM_2.5_	64.9	2000–2015	Cohort study	116,821	≥18	CVD	Mortality	7
Yang et al. (47)	Foshan, China	PM_2.5_	39.2	2015–2019	Cohort study	61,884	≥18	CVD	Morbidity	5
Lin et al. (48)	Taiwan, China	PM_2.5_	32.5	2005–2011	Cohort study	140,911	Infants	RSD	Morbidity	7
Yang et al. (49)	Northern China	PM_2.5_	66.3	–	Cohort study	38,140	≥18	CVD	Mortality	5
Paoin et al. (51)	Thailand	PM_10_	44.4	2005–2013	Cohort study	25,532	≥18	CVD	Morbidity	8
Shi et al. (50)	China	PM_2.5_, PM_10_	52.1/93.0	2016–2018	Cohort study	4,866	Average 65.2	CVD and RSD	Morbidity, mortality	7

CVD, Cardiovascular disease; RSD, Respiratory disease; CHD, Congenital heart disease.

Table 1 presents the risk-of-bias assessments. Twenty-five studies were rated as “low risk.” However, 15 studies were rated as “medium risk” due to inadequate adjustment for potential confounders in the analysis and a lack of exposure assessment, mainly because pollutants were measured once over a large geographical area and not measured at least daily.

### Effects of PM_2.5_ per 10 μg/m^3^ increment on cardiovascular and respiratory diseases

Included in the meta-analysis were 22 cohort studies (22, 23, 25, 26, 28, 30, 33, 35, 36, 38–42, 44–50, 52) and two case-control studies (35, 37) published after 2014 that evaluated the mortality and morbidity attributed to the long-term effects of PM_2.5_.

**Figure 2:**
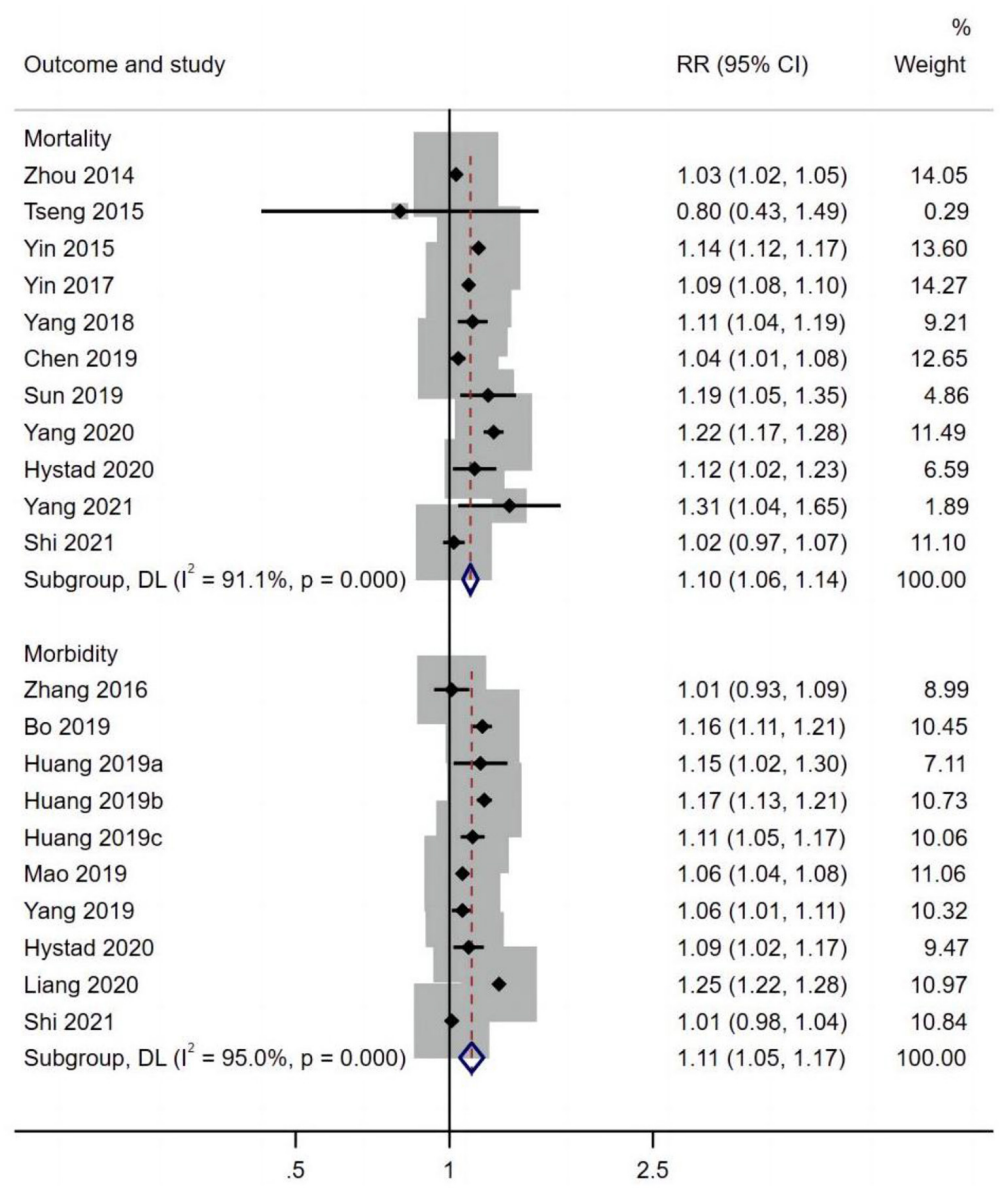
PM_2.5_ effects on cardiovascular disease mortality and morbidity.

**Figure 3:**
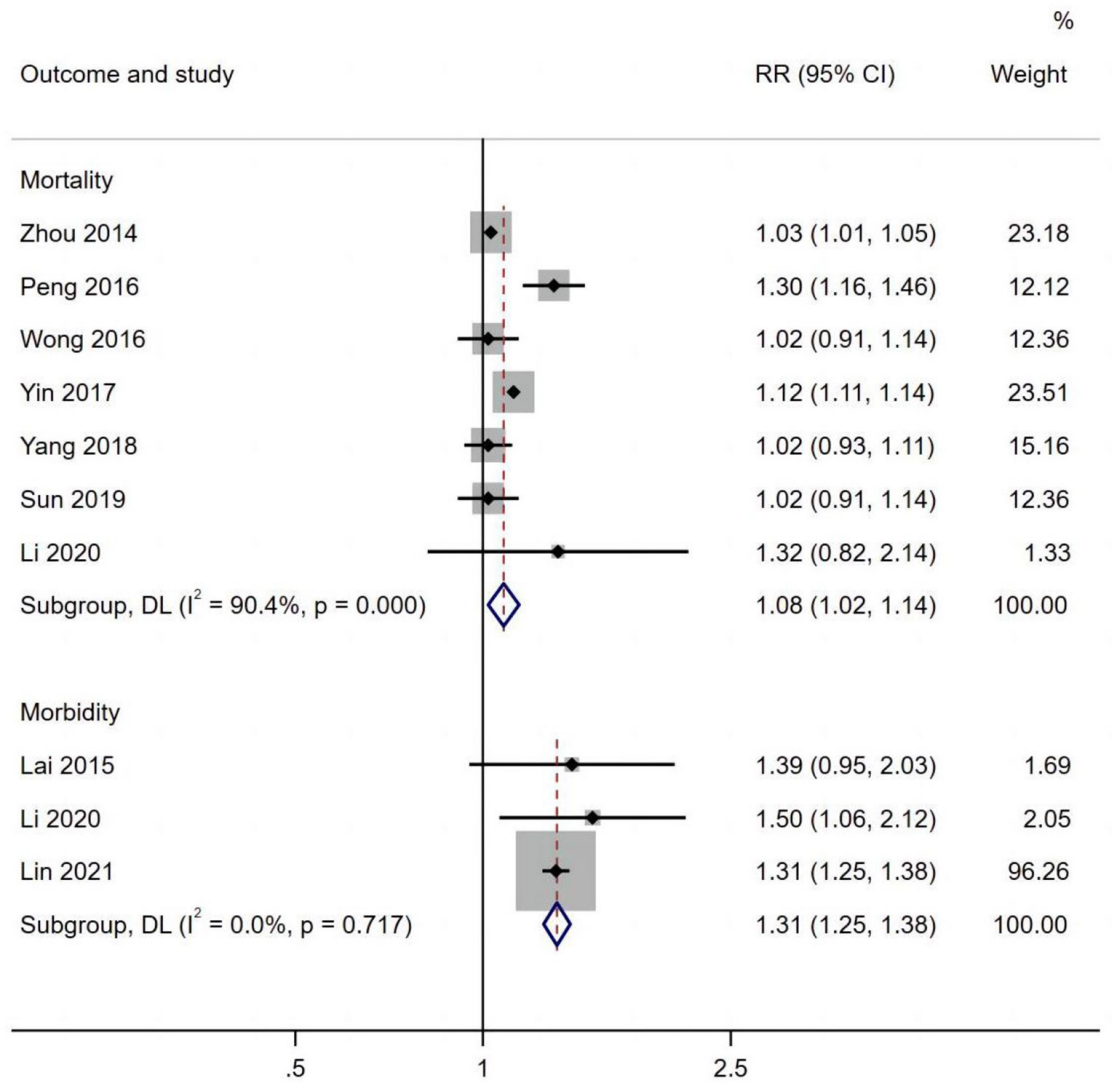
PM_2.5_ effects on respiratory mortality and morbidity.

Figure 2 presents the pooled estimates of the correlation between exposure to PM_2.5_ and cardiovascular disease. Overall, long-term exposure to PM_2.5_ per 10 μg/m^3^ increment was associated with an increased risk of cardiovascular morbidity (RR 1.11, 95% CI: 1.05, 1.17) and mortality (RR 1.10, 95% CI: 1.06, 1.14). Furthermore, exposure to PM_2.5_ per 10 μg/m^3^ increment was significantly associated with an increased risk of respiratory mortality (RR 1.31, 95% CI: 1.25, 1.38) and morbidity (RR 1.08, 95% CI: 1.02, 1.04) (Figure 3).

Table 2 shows that the morbidity of stroke (RR 1.09, 95% CI: 1.06, 1.12) was related to PM_2.5_ exposure per 10 μg/m^3^ increment. A significant association between PM_2.5_ andcardiovascular morbidity was observed in both males (RR 1.08, 95% CI: 1.06, 1.10) and females (RR 1.14, 95% CI: 1.12, 1.17). In addition, the mortality rates for COPD (RR 1.12, 95% CI: 1.11, 1.14), tuberculosis (RR 1.22, 95% CI: 1.09, 1.36), and lung cancer (RR 1.12, 95% CI: 1.09, 1.16) were all associated with long-term exposure to PM_2.5_, and an increased risk of respiratory mortality was observed in elderly persons over 65 years old (RR 1.21, 95% CI: 1.00, 1.47).

**Table 2:** Subgroup analysis of the effects of PM_2.5_ per 10 μg/m^3^ increment on cardiovascular and respiratory diseases.

**Characteristics**	***n***	**RR (95%CI)**	**I^2^**	***P***	***P*-interaction**
**cardiovascular morbidity**					
**Type of study**					
Cohort	8 (28, 35, 36, 41, 42, 45, 47, 50)	1.07 (1.06, 1.09)	99.1	<0.001	0.264
Case-control	1 (34)	1.15 (1.02, 1.30)	-	0.024	
**Sex**					
Male	3 (38, 42, 45)	1.08 (1.06, 1.10)	98.2	<0.001	<0.001
Female	3 (38, 42, 45)	1.14 (1.12, 1.17)	96.4	<0.001	
**Type of disease**					
Stroke	4 (35, 42, 45, 46)	1.09 (1.06, 1.12)	78	<0.001	<0.001
CHD	2 (28, 34)	1.01 (0.96, 1.08)	75.2	0.635	
TF	2 (35, 42)	1.07 (0.92, 1.24)	0	0.379	
Hypertension	2 (36, 41)	1.14 (1.09, 1.19)	35.8	0.212	
**Respiratory mortality**					
**Type of disease**					
COPD	2 (30, 33)	1.12 (1.11, 1.14)	0	<0.001	0.36
Tuberculosis	2 (23, 25)	1.22 (1.09, 1.36)	0	0.001	
Lung cancer	3 (26, 30, 44)	1.12 (1.09, 1.16)	0	<0.001	
**Age**					
≥65	4 (7, 26, 33, 39)	1.21 (1.00, 1.47)	84.6	<0.001	<0.001
<65	3 (25, 30, 44)	1.13 (0.82, 1.55)	55.4	0.106	

CHD, Congenital heart disease; TF, Tetralogy of Fallot; COPD, Chronic obstructive pulmonary disease.

### Effects of PM_10_ per 10 μg/m^3^ increment on cardiovascular and respiratory diseases

**Figure 4:**
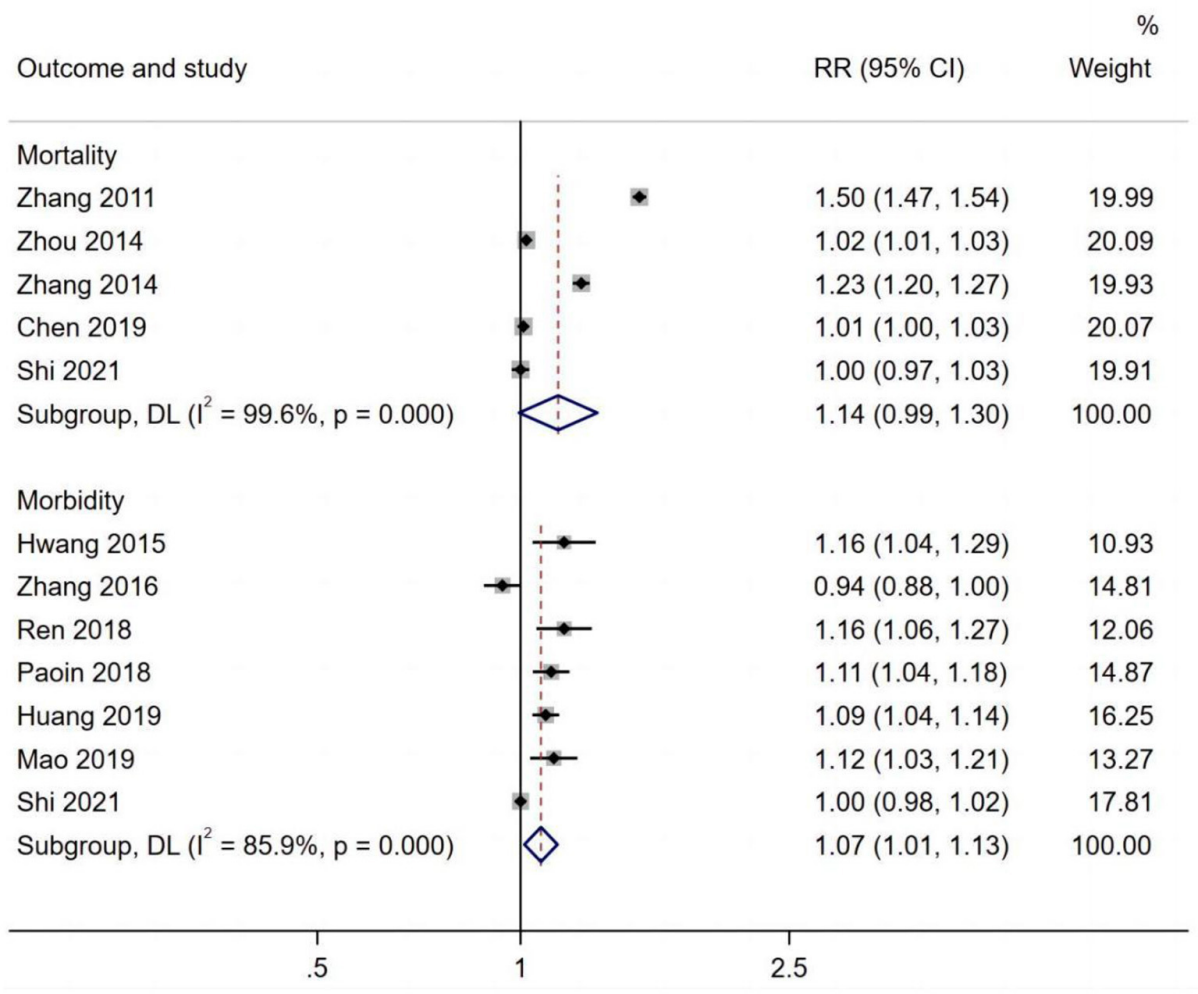
PM_10_ effects on cardiovascular disease mortality and morbidity.

Ten studies assessed the long-term exposure to PM_10_ and cardiovascular diseases. A positive association was observed between PM_10_ andcardiovascular morbidity (RR 1.07, 95% CI 1.01, 1.13) (Figure 4).

**Figure 5:**
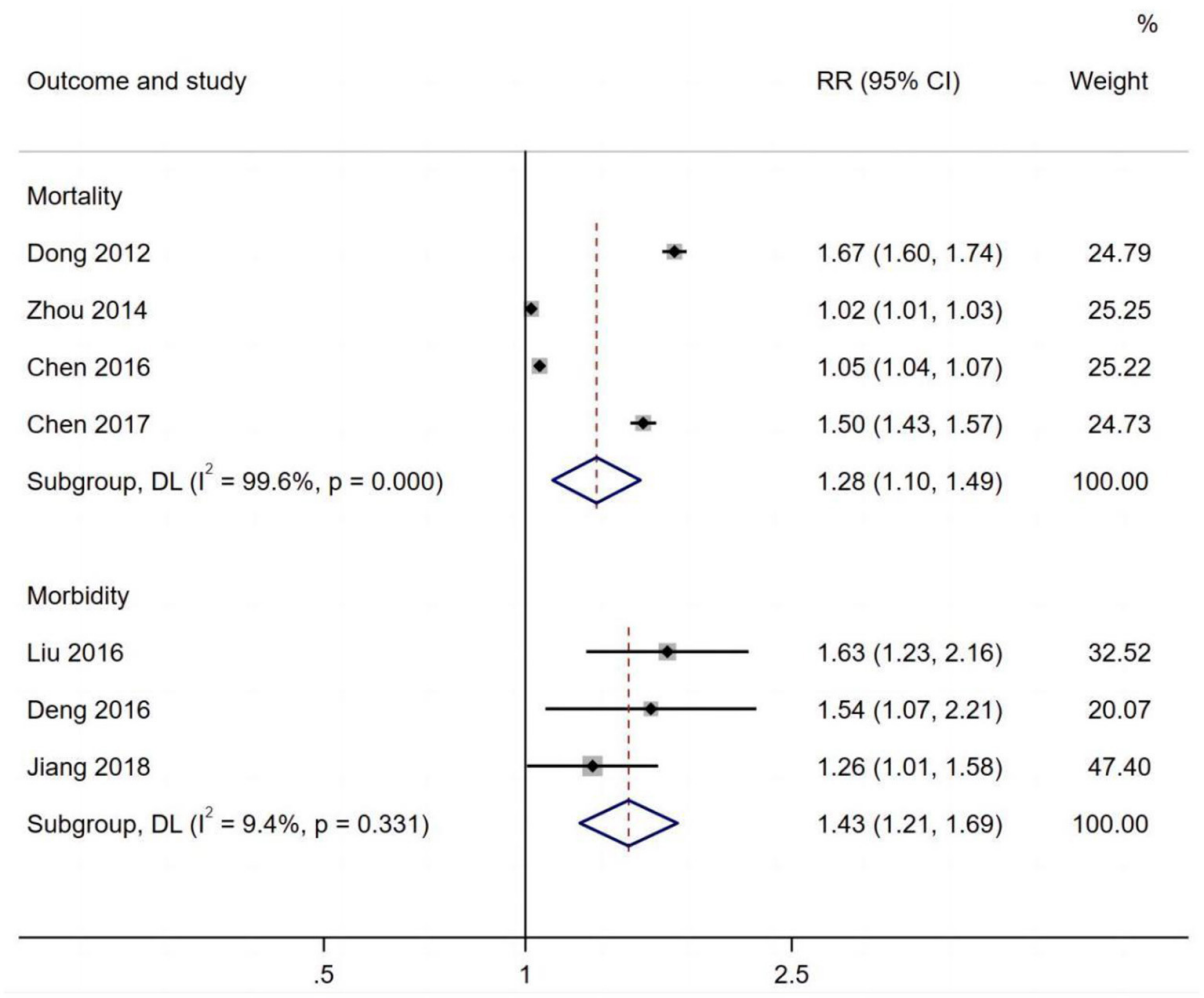
PM_10_ effects on respiratory mortality and morbidity.

Figure 5 shows that respiratory morbidity (RR 1.43, 95% CI: 1.21, 1.69) and mortality (RR 1.28, 95% CI 1.10, 1.49) were both related to long-term exposure to PM_10_.

Subgroup analyses of type of disease, sex, population, and country were performed for morbidity and mortality (Table 3). The risk of VSD morbidity (RR 1.03, 95% CI: 1.02, 1.04) increased per 10 μg/m^3^ increment of PM_10_. A significant association between PM_10_ and cardiovascular mortality was observed for both males (RR 1.06, 95% CI: 1.04, 1.07) and females (RR 1.15, 95% CI: 1.13, 1.17).

**Table 3:** Subgroup analysis of the effects of PM_10_ per 10 μg/m^3^ increment on cardiovascular and respiratory diseases.

**Characteristic**	**n**	**RR (95%CI)**	**I^2^**	***P***	***P*-interaction**
**PM**_10_ **effects on cardiovascular morbidity and mortality**					
**Morbidity**					
**Type of disease**					
CHD	3 (28, 31, 34)	1.02 (0.99, 1.06)	87.5	<0.001	0.386
VSD	3 (21, 28, 35)	1.03 (1.02, 1.04)	98.6	<0.001	
ASD	2 (21, 35)	1.02 (1.01, 1.03)	9.7	0.293	
TF	3 (21, 28, 34)	1.01 (0.99, 1.03)	52.3	0.123	
**Mortality**					
**Sex**					
Male	3 (17, 18, 37)	1.06 (1.04, 1.07)	98.4	<0.001	<0.001
Female	3 (17, 18, 37)	1.15 (1.13, 1.17)	99.5	<0.001	
**PM**_10_ **effects on respiratory morbidity and mortality**					
**Morbidity**					
**Disease**					
Pneumonia	2 (32, 43)	1.01 (1.00, 1.01)	0	0.358	0.013
AR	1 (27)	3.34 (1.42, 7.88)	0	-	
Asthma	1 (24)	0.85 (0.62, 1.16)	0	-	
**Mortality**					
**Disease**					
COPD	2 (6, 29)	1.02 (0.99, 1.06)	95.3	<0.001	0.443
Lung cancer	2 (6, 7)	1.04 (1.02, 1.06)	99.3	<0.001	

CHD, Congenital heart disease; VSD, Ventricular septal defect; ASD, Atrial septal defect; TF, Tetralogy of Fallot; AR, Allergic rhinitis; COPD, Chronic obstructive pulmonary disease.

Very few studies of the effects of long-term exposure to PM_10_ on respiratory diseases have been carried out. Two studies assessed long-term exposure to PM_10_ and lung cancer, and a positive association was observed (RR 1.04, 95% CI: 1.02, 1.06).

### Effects of PM_1_ per 10 μg/m^3^ increment on cardiovascular diseases

Two studies (29, 41) assessed long-term exposure to PM_1_ and cardiovascular diseases. One study assessed long-term exposure to PM_1_ per 10 μg/m^3^ increment and cardiovascular disease mortality, and a positive association was observed (RR 1.06, 95% CI: 1.03, 1.10). Another study assessed long-term exposure to PM_1_ per 10 μg/m^3^ increment andcardiovascular morbidity and similarly found a positive association (RR 1.11, 95% CI: 1.01, 1.22). A forest plot of the effects of PM_1_ on cardiovascular diseases can be found in the online Supplementary material.

### Publication bias

Egger's test (*p* = 0. 0.615, *n* = 11) was conducted for the literature regarding the effects of PM_2.5_ on cardiovascular disease mortality, and no publication bias was found. Publication bias was not assessed for PM_10_ and PM_1_ because fewer than 10 studies were considered in the meta-analysis.

## Discussion

To the best of our knowledge, this is the first systematic review and meta-analysis to explore the effects of long-term exposure to particulate matter on the morbidity and mortality of cardiovascular and respiratory diseases in LMICs. Increased risk for CVD and respiratory disease events was associated with increased PM_2.5_ and PM_10_ concentrations. The results regarding the effects of PM_1_ were not remarkable because of the small sample size; thus, more studies are needed.

PM pollution has considerable effects on the cardiovascular and respiratory systems. Atmospheric PM exposure, even at very low concentrations, could seriously affect the health of humans. Due to its small particle size, PM_1_ stays in the atmosphere for a long time; these particles have a long transport distance and comprise many harmful and toxic substances, such as polycyclic aromatic hydrocarbons, which can have harmful human health effects. Although its effects on health are significant (53, 54), few studies focus on PM_1_ pollution. In contrast, there are many studies about the effects of PM_2.5_ and PM_10_ on human health (55, 56). Some studies have shown that PM_10_ is made up of fine (PM_2.5_) and coarse particles. Unfortunately, the presented effect estimates for PM_2.5_ and PM_10_ cannot be compared as the applied increment of 10 μg/m^3^ represents a larger contrast for PM_2.5_ than PM_10_. The biological mechanisms by which PM affects cardiovascular health include metabolic activation, oxidative stress, genotoxicity, inflammation, and autophagy interference (57). Cells involved in these physiological and biochemical processes affect cardiovascular and respiratory system functions in target cells and result in pathophysiological changes, such as cardiac autonomic nervous system adjustments, high blood pressure, metabolic disorders, atherosclerosis and deterioration, inflammatory injury, mutagenicity, and airway epithelial defense function defects, eventually leading to a series of cardiovascular and respiratory events and even death.

This pattern is consistent with the findings of previous studies. Momtazan et al.'s results showed that high levels of particulate matter in the air drastically increased the number of people with cardiovascular diseases (58). In Chen and Hoek (15), a systematic review and meta-analysis evaluated long-term exposure to PM and all-cause and cause-specific mortality; clear evidence showed that both PM_2.5_ and PM_10_ were associated with increased all cause, cardiovascular disease, and respiratory mortality, but PM_2.5_ had a greater effect than PM_10_, especially on respiratory diseases. In this study, the combined risk ratio (RR) for PM_2.5_ and respiratory mortality in LMICs was 1.31, (95% CI: 1.25, 1.38) per 10 μg/m^3^ increase; compared with Chen's study, this result is significantly higher than the research results for the global area (RR 1.10, 95% CI: 1.03, 1.18) and even higher than those of high-income countries (RR 1.04, 95% CI: 1.03, 1.06).

Ambient particulate matter air pollution has increasingly significant effects on health in LMICs. Compared to those in high-income countries, populations in LMICs are burdened with a greater proportion of PM, leading to their extensive distribution as anthropogenic PM increases (59). Management of PM pollution is a challenging process, especially for LMICs with serious economic and health resource problems. Compared to that of the last WHO global assessment, the evidence available has increased considerably (60–66); nevertheless, studies carried out in LMICs remain rare. Studies on the effects of PM on the health of LMICs' populations are scarce. LMICs may have published relevant articles in their own national languages, but the language issue prevents many of these studies from being included. Through systematic analysis, we can obtain effect data for LMICs to provide support for the formulation of appropriate improvement policies. These findings are essential to inform policymakers and eventually alleviate the burden of PM ambient air pollution in LMICs.

### Strengths and limitations

This systematic review and meta-analysis provide comprehensive and current evidence of the effects of long-term exposure to particulate matter on the morbidity and mortality of cardiovascular and respiratory diseases in LMICs. However, our study has some limitations. First, significant heterogeneity for the pooled estimates was noted in the meta-analysis; this finding might be due to the high variability in the study populations, outcomes, and geographical locations. Therefore, subgroup analyses of sex (male vs. female), population age (< 65 years vs. >65 years), study type (cohort study vs. case-control), and disease type were conducted to further investigate the potential contributing sources. Second, most of the papers included in our study were from China; this parameter affects the pooled estimates, although it is an inherent and inevitable selection bias. Third, we found that relatively few studies were performed in LMICs. The earliest included studies of the chronic effects of PM pollution on respiratory and cardiovascular diseases were reported in 2011. Results from individual studies may not be representative, and the limited sample size cannot yield statistically significant conclusions. To support health effects assessments in LMICs and global burden of disease assessments, new studies in LMICs are needed.

### Suggestions for further research

First, the present evidence regarding long-term exposure to particulate matter in LMICs was mainly from China. Studies assessing the effects in other geographical locations are suggested and could contribute to the evaluation of the potentially different effects of particulate matter on different continents. Second, PM_1_ is the smallest particle, and its health effects should not be understated. Future studies should monitor the chronic effects of PM_1_ on health status for longer. Third, a greater number of studies are needed to prove the association between long-term exposure to particulates and cardiovascular and respiratory diseases in vulnerable populations; special attention should be paid to the relationship between long-term exposure to particulates and pregnant women, newborn diseases, mental disorders, and infectious diseases.

## Conclusions

Long-term exposure to PM_2.5_, PM_10_, and PM_1_ was all related to cardiovascular and respiratory disease events. PM_2.5_ had a greater effect than PM_10_, especially on respiratory diseases, and the risk of respiratory mortality was significantly higher for LMICs than high-income countries. More studies are needed to confirm the effect of PM_1_ on cardiovascular and respiratory diseases.

## Data availability statement

The original contributions presented in the study are included in the article/Supplementary material, further inquiries can be directed to the corresponding authors.

## Author contributions

Criteria setting were performed by XS and XH. The data were collected by JG, XF, and ZL. The first draft of the manuscript was written by JG. GC and KY were the instructors. All authors contributed to the study conception, design, read, and approved the final manuscript.

## References

[1] Hamra GB Guha N Cohen A Laden F Raaschou-Nielsen O Samet JM. Outdoor particulate matter exposure and lung cancer: a systematic review and meta-analysis. Environ Health Perspect. (2014) 122:906–11. 10.1289/ehp/1408092 24911630 PMC4154221

[2] Pope CA Dockery DW. Health effects of fine particulate air pollution: lines that connect. J Air Waste Manag Assoc (1995). (2006) 56:709–42. 10.1080/10473289.2006.10464485 16805397

[3] Brunekreef B Holgate ST. Air pollution and health. Lancet (London, England). (2002) 360:1233–42. 10.1016/S0140-6736(02)11274-8 12401268

[4] Samoli E Analitis A Touloumi G Schwartz J Anderson HR Sunyer J. Estimating the exposure-response relationships between particulate matter and mortality within the APHEA multicity project. Environ Health Perspect. (2005) 113:88–95. 10.1289/ehp.7387 15626653 PMC1253715

[5] Braam LA Bushnell DM Martin ML Pierson RF. Systematic review of patient-reported outcome measures used to assess symptoms associated with heart failure. Appl Res Qual Life. (2015) 24:78–79.

[6] Chen X Zhang LW Huang JJ Song FJ Zhang LP Qian ZM. Long-term exposure to urban air pollution and lung cancer mortality: a 12-year cohort study in Northern China. Sci Total Environ. (2016) 571:855–61. 10.1016/j.scitotenv.2016.07.064 27425436

[7] Zhou M Liu Y Wang L Kuang X Xu X Kan H. Particulate air pollution and mortality in a cohort of Chinese men. Environ Pollut. (2014) 186:1–6. 10.1016/j.envpol.2013.11.010 24333659

[8] Liu C Chen R Sera F Vicedo-Cabrera AM Guo Y Tong S. Ambient particulate air pollution and daily mortality in 652 cities. N Engl J Med. (2019) 381:705–15. 10.1056/NEJMoa1817364 31433918 PMC7891185

[9] Jiang S Yu ZG Anh VV Zhou Y. Long- and short-term time series forecasting of air quality by a multi-scale framework. Environ Pollut. (2021) 271:116381. 10.1016/j.envpol.2020.116381 33421843

[10] Shah AS Langrish JP Nair H McAllister DA Hunter AL Donaldson K. Global association of air pollution and heart failure: a systematic review and meta-analysis. Lancet (London, England). (2013) 382:1039–48. 10.1016/S0140-6736(13)60898-3 23849322 PMC3809511

[11] Linn WS Szlachcic Y Gong H Jr. Kinney PL Berhane KT. Air pollution and daily hospital admissions in metropolitan Los Angeles. Environ Health Perspect. (2000) 108:427–34. 10.1289/ehp.00108427 10811569 PMC1638060

[12] Li Y Cao L Zhang Z Hou L Qin Y Hui X. Reporting and methodological quality of COVID-19 systematic reviews needs to be improved: an evidence mapping. J Clin Epidemiol. (2021) 135:17–28. 10.1016/j.jclinepi.2021.02.021 33657455 PMC8313077

[13] Song X Hu Y Ma Y Jiang L Wang X Shi A. Is short-term and long-term exposure to black carbon associated with cardiovascular and respiratory diseases? A systematic review and meta-analysis based on evidence reliability. BMJ open. (2022) 12:e049516. 10.1136/bmjopen-2021-049516 35504636 PMC9066484

[14] Cumpston M Li T Page MJ Chandler J Welch VA Higgins JP. Updated guidance for trusted systematic reviews: a new edition of the Cochrane Handbook for Systematic Reviews of Interventions. Cochrane Database Syst Rev. (2019) 10:ED000142. 10.1002/14651858.ED000142 31643080 PMC10284251

[15] Chen J Hoek G. Long-term exposure to PM and all-cause and cause-specific mortality: a systematic review and meta-analysis. Environ Int. (2020) 143:105974. 10.1016/j.envint.2020.105974 32703584

[16] Lu F Xu D Cheng Y Dong S Guo C Jiang X. Systematic review and meta-analysis of the adverse health effects of ambient PM25 and PM10 pollution in the Chinese population. Environ Res. (2015) 136:196–204. 10.1016/j.envres.2014.06.029 25460637

[17] Zhang P Dong G Sun B Zhang L Chen X Ma N. Long-term exposure to ambient air pollution and mortality due to cardiovascular disease and cerebrovascular disease in Shenyang, China. PLoS ONE. (2011) 6:e20827. 10.1371/journal.pone.0020827 21695220 PMC3112212

[18] Zhang LW Chen X Xue XD Sun M Han B Li CP. Long-term exposure to high particulate matter pollution and cardiovascular mortality: a 12-year cohort study in four cities in northern China. Environ Int. (2014) 62:41–7. 10.1016/j.envint.2013.09.012 24161381

[19] Dong GH Zhang P Sun B Zhang L Chen X Ma N. Long-term exposure to ambient air pollution and respiratory mortality in Shenyang, China: a 12-year population-based retrospective cohort study. Respiration. (2012) 84:360–8. 10.1159/000332930 22116521

[20] Jin L Qiu J Zhang Y Qiu W He X Wang Y. Ambient air pollution and congenital heart defects in Lanzhou, China. Environ Res Lett. (2015) 10:074005. 10.1088/1748-9326/10/7/074005 31555342 PMC6760856

[21] Hwang BF Lee YL Jaakkola JJ. Air pollution and the risk of cardiac defects: a population-based case-control study. Medicine. (2015) 94:e1883. 10.1097/MD.0000000000001883 26554783 PMC4915884

[22] Yin P Brauer M Cohen A Burnett RT Liu JM Liu YN. Ambient fine particulate matter exposure and cardiovascular mortality in China: a prospective cohort study. Lancet (London, England). (2015) 386:6. 10.1016/S0140-6736(15)00584-X

[23] Lai TC Chiang CY Wu CF Yang SL Liu DP. Ambient air pollution and risk of tuberculosis: a cohort study. Occup Environ Med. (2016) 73:56–61. 10.1136/oemed-2015-102995 26514394

[24] Liu W Huang C Hu Y Fu Q Zou Z Sun C. Associations of gestational and early life exposures to ambient air pollution with childhood respiratory diseases in Shanghai, China: a retrospective cohort study. Environ. Int. (2016) 92–93:284–93. 10.1016/j.envint.2016.04.019 27128713

[25] Peng Z Liu C Xu B Kan H Wang W. Long-term exposure to ambient air pollution and mortality in a Chinese tuberculosis cohort. Sci Total Environ. (2017) 580:1483–8. 10.1016/j.scitotenv.2016.12.128 28038878

[26] Wong CM Tsang H Lai HK Thomas GN Lam KB Chan KP. Cancer Mortality risks from long-term exposure to ambient fine particle. Cancer Epidemiol Biomarkers Prev. (2016) 25:839–45. 10.1158/1055-9965.EPI-15-0626 27197138 PMC5505442

[27] Deng Q Lu C Yu Y Li Y Sundell J Norback D. Early life exposure to traffic-related air pollution and allergic rhinitis in preschool children. Respir Med. (2016) 121:67–73. 10.1016/j.rmed.2016.10.016 27888994

[28] Zhang B Liang S Zhao J Qian Z Bassig BA Yang R. Maternal exposure to air pollutant PM25 and PM10 during pregnancy and risk of congenital heart defects. J Expo Sci Environ Epidemiol. (2016) 26:422–7. 10.1038/jes.2016.1 26883477 PMC4913168

[29] Chen X Wang X Huang JJ Zhang LW Song FJ Mao HJ. Nonmalignant respiratory mortality and long-term exposure to PM(10) and SO(2): a 12-year cohort study in northern China. Environ. Pollut. (2017) 231:761–67. 10.1016/j.envpol.2017.08.085 28865381

[30] Yin P Brauer M Cohen A Burnett RT Liu J Liu Y. Long-term fine particulate matter exposure and nonaccidental and cause-specific mortality in a large national cohort of Chinese men. Environ Health Perspect. (2017) 125:117002. 10.1289/EHP1673 29116930 PMC5947939

[31] Ren Z Zhu J Gao Y Yin Q Hu M Dai L. Maternal exposure to ambient PM(10) during pregnancy increases the risk of congenital heart defects: evidence from machine learning models. Sci Total Environ. (2018) 630:1–10. 10.1016/j.scitotenv.2018.02.181 29471186

[32] Jiang W Lu C Miao YF Xiang YG Chen L Deng QH. Outdoor particulate air pollution and indoor renovation associated with childhood pneumonia in China. Atmos Environ. (2018) 174:76–81. 10.1016/j.atmosenv.2017.11.043

[33] Yang Y Tang R Qiu H Lai PC Wong P Thach TQ. Long term exposure to air pollution and mortality in an elderly cohort in Hong Kong. Environ Int. (2018) 117:99–106. 10.1016/j.envint.2018.04.034 29730535

[34] Huang CC Chen BY Pan SC Ho YL Guo YL. Prenatal exposure to PM(25) and congenital heart diseases in Taiwan. Sci Total Environ. (2019) 655:880–6. 10.1016/j.scitotenv.2018.11.284 30481714

[35] Huang K Liang F Yang X Liu F Li J Xiao Q. Long term exposure to ambient fine particulate matter and incidence of stroke: prospective cohort study from the China-PAR project. BMJ (Clinical research ed). (2019) 367:l6720. 10.1136/bmj.l6720 31888885 PMC7190010

[36] Huang K Yang X Liang F Liu F Li J Xiao Q. Long-term exposure to fine particulate matter and hypertension incidence in China. Hypertension (Dallas, Tex : 1979). (2019) 73:1195–201. 10.1161/HYPERTENSIONAHA.119.12666 31067193 PMC6656583

[37] Chen G Wang A Li S Zhao X Wang Y Li H. Long-term exposure to air pollution and survival after ischemic stroke. Stroke. (2019) 50:563–70. 10.1161/STROKEAHA.118.023264 30741622 PMC6389419

[38] Mao S Chen G Liu F Li N Wang C Liu Y. Long-term effects of ambient air pollutants to blood lipids and dyslipidemias in a Chinese rural population. Environ Poll (Barking, Essex : 1987). (2020) 256:113403. 10.1016/j.envpol.2019.113403 31711721

[39] Sun S Cao W Qiu H Ran J Lin H Shen C. Benefits of physical activity not affected by air pollution: a prospective cohort study. Int J Epidemiol. (2020) 49:142–52. 10.1093/ije/dyz184 31504557

[40] Yang BY Guo Y Morawska L Bloom MS Markevych I Heinrich J. Ambient PM(1) air pollution and cardiovascular disease prevalence: Insights from the 33 Communities Chinese Health Study. Environ Int. (2019) 123:310–7. 10.1016/j.envint.2018.12.012 30557810

[41] Bo Y Guo C Lin C Chang LY Chan TC Huang B. Dynamic changes in long-term exposure to ambient particulate matter and incidence of hypertension in adults. Hypertension (Dallas, Tex : 1979). (2019) 74:669–77. 10.1161/HYPERTENSIONAHA.119.13212 31303109

[42] Hystad P Larkin A Rangarajan S AlHabib KF Avezum A Calik KBT. Associations of outdoor fine particulate air pollution and cardiovascular disease in 157 436 individuals from 21 high-income, middle-income, and low-income countries (PURE): a prospective cohort study. Lancet Planetary health. (2020) 4:e235–45. 10.1016/S2542-5196(20)30103-0 32559440 PMC7457447

[43] Ruchiraset A Tantrakarnapa K. Association of climate factors and air pollutants with pneumonia incidence in Lampang province, Thailand: findings from a 12-year longitudinal study. Int J Environ Health Res. (2022) 32:691–700. 10.1080/09603123.2020.1793919 32662678

[44] Li J Lu X Liu F Liang F Huang K Yang X. Chronic effects of high fine particulate matter exposure on lung cancer in China. Am J Respir Crit Care Med. (2020) 202:1551–9. 10.1164/rccm.202001-0002OC 32614242 PMC8168622

[45] Liang F Liu F Huang K Yang X Li J Xiao Q. Long-term exposure to fine particulate matter and cardiovascular disease in China. J Am Coll Cardiol. (2020) 75:707–17. 10.1016/j.jacc.2019.12.031 32081278

[46] Yang X Liang F Li J Chen J Liu F Huang K. Associations of long-term exposure to ambient PM(25) with mortality in Chinese adults: A pooled analysis of cohorts in the China-PAR project. Environm. Int. (2020) 138:105589. 10.1016/j.envint.2020.105589 32146266 PMC8164211

[47] Yang Y Lin Q Liang Y Ruan Z Acharya BK Zhang S. Maternal air pollution exposure associated with risk of congenital heart defect in pre-pregnancy overweighted women. Sci Total Environ. (2020) 712:136470. 10.1016/j.scitotenv.2019.136470 31931190

[48] Lin YT Shih H Jung CR Wang CM Chang YC Hsieh CY. Effect of exposure to fine particulate matter during pregnancy and infancy on paediatric allergic rhinitis. Thorax. (2021) 76:568–74. 10.1136/thoraxjnl-2020-215025 33707186

[49] Yang X Zhang L Chen X Liu F Shan A Liang F. Long-term exposure to ambient PM(25) and stroke mortality among urban residents in northern China. Ecotoxicol Environ Saf. (2021) 213:112063. 10.1016/j.ecoenv.2021.112063 33636465 PMC8150861

[50] Shi Y Zhang L Li W Wang Q Tian A Peng K. Association between long-term exposure to ambient air pollution and clinical outcomes among patients with heart failure: Findings from the China PEACE Prospective Heart Failure Study. Ecotoxicol Environ Saf. (2021) 222:112517. 10.1016/j.ecoenv.2021.112517 34303044

[51] Paoin K Ueda K Ingviya T Buya S Phosri A Seposo XT. Long-term air pollution exposure and self-reported morbidity: a longitudinal analysis from the Thai cohort study (TCS). Environ Res. (2021) 192:110330. 10.1016/j.envres.2020.110330 33068582 PMC7768181

[52] Tseng E Ho WC Lin MH Cheng TJ Chen PC Lin HH. Chronic exposure to particulate matter and risk of cardiovascular mortality: cohort study from Taiwan. BMC Public Health. (2015) 15:936. 10.1186/s12889-015-2272-6 26392179 PMC4578246

[53] Guo HG Li X Li WF Wu JS Wang SY Wei J. Climatic modification effects on the association between PM1 and lung cancer incidence in China. BMC Public Health. (2021) 21:880. 10.1186/s12889-021-10912-8 33962607 PMC8106137

[54] Chen G Li S Zhang Y Zhang W Daowei L Wei X. Effects of ambient PM 1 air pollution on daily emergency hospital visits in China: an epidemiological study. Lancet Planetary Health. (2017) 1:e221–e229. 10.1016/S2542-5196(17)30100-6 29851607

[55] Xing YF Xu YH Shi MH Lian YX. The impact of PM25 on the human respiratory system. J Thorac Dis. (2016) 8:E69–74. 10.3978/j.issn.2072-1439.2016.01.19 26904255 PMC4740125

[56] Yang H Peng Q Zhou J Song G. Gong X. The unidirectional causality influence of factors on PM(25) in Shenyang city of China. Sci Rep. (2020) 10:8403. 10.1038/s41598-020-65391-5 32439904 PMC7242410

[57] Hamanaka RB Mutlu GM. Particulate matter air pollution: effects on the cardiovascular system. Front Endocrinol (Lausanne). (2018) 9:680. 10.3389/fendo.2018.00680 30505291 PMC6250783

[58] Momtazan M Geravandi S Rastegarimehr B Valipour A Ranjbarzadeh A Yari AR. An investigation of particulate matter and relevant cardiovascular risks in Abadan and Khorramshahr in 2014–2016. Toxin Rev. (2019) 38:290–7. 10.1080/15569543.2018.1463266

[59] Newell K Kartsonaki C Lam KBH Kurmi OP. Cardiorespiratory health effects of particulate ambient air pollution exposure in low-income and middle-income countries: a systematic review and meta-analysis. The Lancet Planetary health. (2017) 1:e368–80. 10.1016/S2542-5196(17)30166-3 29851649

[60] Tahery N Geravandi S Goudarzi G Shahriyari HA Jalali S Mohammadi MJ. Estimation of PM(10) pollutant and its effect on total mortality (TM), hospitalizations due to cardiovascular diseases (HACD), and respiratory disease (HARD) outcome. Environ Sci Pollut Res Int. (2021) 28:22123–30. 10.1007/s11356-020-12052-9 33411285

[61] Faraji Ghasemi F Dobaradaran S Saeedi R Nabipour I Nazmara S Ranjbar Vakil Abadi D. Levels and ecological and health risk assessment of PM2.5-bound heavy metals in the northern part of the Persian Gulf. Environ Sci Pollut Res Int. (2019) 27:5305–13. 10.1007/s11356-019-07272-7 31848967

[62] Moradi M Mokhtari A Mohammadi MJ Hadei M. Vosoughi M. Estimation of long-term and short-term health effects attributed to PM(25) standard pollutants in the air of Ardabil (using Air Q + model). Environ Sci Pollut Res. (2022) 29:21508–16. 10.1007/s11356-021-17303-x 34761318

[63] Borsi SH Goudarzi G Sarizadeh G Dastoorpoor M Geravandi S Shahriyari HA. Health endpoint of exposure to criteria air pollutants in ambient air of on a populated in Ahvaz City, Iran. Frontiers in public health. (2022) 10:869656. 10.3389/fpubh.2022.869656 35425736 PMC9002232

[64] Effatpanah M Effatpanah H Jalali S Parseh I Goudarzi G. Hospital admission of exposure to air pollution in Ahvaz megacity during 2010–2013. Clin Epidemiology Glo. (2020) 8:550–56. 10.1016/j.cegh.2019.12.001

[65] Khaefi M Goudarzi G Yari A Geravandi S Dobaradaran S. An association between ambient pollutants and hospital admitted respiratory cases in Ahvaz, Iran. Fresenius Environ Bull. (2016) 25:3955–61.

[66] Morovati P Valipour A Geravandi S Karimyan A Adeli R Mohammadi M. Association of air quality index related to criteria air pollutants in Abadan, Iran. Fresenius Environ Bull. (2018) 27:4023–8.

