# Supplementary material for: Long-term exposure to particulate matter on cardiovascular and respiratory diseases in low- and middle-income countries: A systematic review and meta-analysis
Source: Front Public Health. 2023 Mar 28;11:1134341. doi: 10.3389/fpubh.2023.1134341 (PMC10089304; doi:10.3389/fpubh.2023.1134341)

Supplementary Materials

Long-term exposure to particulate matter on cardiovascular and respiratory diseases in low-and middle-income countries: a systematic review and meta-analysis

Supplemental 1 : Search strategy

	**Embase**	
No.	Search Details	Results
1	'air pollution'/exp OR 'particulate matter'/exp	185,675
2	'air pollut*':ab,ti OR 'air contamination*':ab,ti OR 'particulate matter*':ab,ti OR pm10:ab,ti OR pm2.5:ab,ti OR particle*:ab,ti OR particulat*:ab,ti OR pm1:ab,ti OR (pm:ab,ti AND 1:ab,ti)	493,777
3	#1 OR #2	601,771
4	'morbidity'/exp OR 'hospitalization'/exp OR 'death'/exp OR 'mortality'/exp OR 'outpatient'/exp	2,223,886
5	morbidit*:ab,ti OR hospitalisation*:ab,ti OR hospitalization*:ab,ti OR death*:ab,ti OR mortalit*:ab,ti OR outpatien*:ab,ti OR 'emergency room*':ab,ti OR 'emergency department*':ab,ti OR 'emergency admi*':ab,ti OR 'hospital admission*':ab,ti	2,868,131
6	#4 OR #5	3,603,063
7	'case control':ab,ti	166,094
8	cohort:ab,ti OR 'follow up':ab,ti OR longitudinal:ab,ti OR prospective:ab,ti OR retrospective:ab,ti	3,559,775
9	'case control study'/exp	178,583
10	'cohort studies'/exp	625,587
11	#7 OR #8 OR #9 OR #10	3,816,779
12	'respiratory tract disease'/exp	2,747,612
13	respirat*:ab,ti OR 'pulmonary disease*':ab,ti OR lung:ab,ti OR 'chest infection*':ab,ti OR airway:ab,ti OR asthma*:ab,ti OR pneumonia*:ab,ti OR 'chronic obstructive pulmonary disease':ab,ti OR copd:ab,ti	1,922,167

14	'cardiovascular diseases'/exp	4,489,132
15	cardio*:ab,ti OR cardiop*:ab,ti OR cardior*:ab,ti OR heart:ab,ti OR coronary:ab,ti OR vascular:ab,ti OR blood:ab,ti OR cardiac:ab,ti	5,165,941
16	#12 OR #13 OR #14 OR #15	9,576,255
17	'developing country'/exp	95,296
18	('afghanistan':ab,ti OR 'guinea':ab,ti OR 'rwanda':ab,ti OR senegal:ab,ti OR 'benin':ab,ti OR 'guinea-bissau':ab,ti OR 'senegal':ab,ti OR 'burkina faso':ab,ti OR 'haiti':ab,ti OR 'sierra leone':ab,ti OR 'burundi':ab,ti OR 'korea dem. people rep*':ab,ti OR 'somalia':ab,ti) AND 'central african republic':ab,ti OR 'liberia':ab,ti OR 'south sudan':ab,ti OR 'chad':ab,ti OR madagascar:ab,ti OR 'tanzania':ab,ti OR 'comoros':ab,ti OR 'malawi':ab,ti OR 'togo':ab,ti OR 'congo dem. rep':ab,ti OR 'mali':ab,ti OR 'uganda':ab,ti OR 'eritrea':ab,ti OR 'mozambique':ab,ti OR 'zimbabwe':ab,ti OR 'ethiopia':ab,ti OR 'nepal':ab,ti OR 'gambia':ab,ti OR 'niger':ab,ti OR 'armenia':ab,ti OR 'kiribati':ab,ti OR 'soloman islands':ab,ti OR 'bangladesh':ab,ti OR 'kosovo':ab,ti OR 'sri lanka':ab,ti OR 'bhutan':ab,ti OR 'kyrgyz*':ab,ti OR 'sudan':ab,ti OR 'bolivia':ab,ti OR 'lao':ab,ti OR 'swaziland':ab,ti OR 'cabo verde':ab,ti OR 'lesotho':ab,ti OR 'syria*':ab,ti OR 'cambodia':ab,ti OR 'mauritania':ab,ti OR 'tajikistan':ab,ti OR 'cameroon':ab,ti OR 'micronesia':ab,ti OR 'timor-leste':ab,ti OR 'congo. rep':ab,ti OR 'moldova':ab,ti OR 'tonga':ab,ti OR 'mongolia':ab,ti OR 'tunisia':ab,ti OR 'dijibouti':ab,ti OR 'morocco':ab,ti OR 'ukraine':ab,ti OR 'egypt, arab rep':ab,ti OR 'myanmar':ab,ti OR 'uzbekistan':ab,ti OR ((('el salvador':ab,ti OR 'nicaragua':ab,ti OR 'vanuatu':ab,ti OR 'ghana':ab,ti OR 'nigeria':ab,ti OR 'vietnam':ab,ti OR 'guatemala':ab,ti OR 'pakistan':ab,ti OR 'west bank':ab,ti) AND gaza:ab,ti OR 'honduras':ab,ti OR 'papua new guinea':ab,ti OR 'yemen*':ab,ti OR 'india':ab,ti OR 'philippines':ab,ti OR 'zambia':ab,ti OR indonesia:ab,ti OR 'samoa':ab,ti OR 'kenya':ab,ti OR 'sao tome':ab,ti) AND principe:ab,ti) OR 'albania':ab,ti OR 'ecuador':ab,ti OR 'montenegro':ab,ti OR (('algeria':ab,ti OR 'fiji':ab,ti OR 'namibia':ab,ti OR 'american samoa':ab,ti OR 'gabon':ab,ti OR 'palau':ab,ti OR 'angola':ab,ti OR 'georgia':ab,ti OR 'panama':ab,ti OR 'argentina':ab,ti OR 'grenada':ab,ti OR 'paraguay':ab,ti OR 'azerbaijan':ab,ti OR 'guyana':ab,ti OR 'peru':ab,ti OR 'belarus':ab,ti OR 'iran, islamic rep':ab,ti OR 'romania':ab,ti OR 'belize':ab,ti OR 'iraq':ab,ti OR 'russia*':ab,ti OR bosnia:ab,ti) AND herzegovina:ab,ti) OR 'jamaica':ab,ti OR 'serbia':ab,ti OR (('botswana':ab,ti OR 'jordan':ab,ti OR 'southafrica':ab,ti OR 'brazil':ab,ti OR 'kazakhstan':ab,ti OR 'st. lucia':ab,ti OR 'bulgaria':ab,ti OR 'lebanon':ab,ti OR 'st vincent':ab,ti) AND 'the grenadines':ab,ti) OR 'china':ab,ti OR 'libya':ab,ti OR	80,9617

	'suriname':ab,ti OR 'colombia':ab,ti OR 'macedonia':ab,ti OR 'thailand':ab,ti OR 'costa rica':ab,ti OR 'malaysia':ab,ti OR 'turkey':ab,ti OR 'cuba':ab,ti OR 'maldives':ab,ti OR 'turkmenistan':ab,ti OR 'dominica':ab,ti OR 'marshall islands':ab,ti OR 'tuvalu':ab,ti OR 'dominican republic':ab,ti OR 'mauritius':ab,ti OR 'venezuela':ab,ti OR 'equatorial guinea':ab,ti OR 'mexico':ab,ti OR 'africa':ab,ti OR 'caribbean region':ab,ti OR 'central america':ab,ti OR 'latinamerica':ab,ti OR 'south america':ab,ti OR 'asia':ab,ti	
19	#17 OR #18	88,4654
20	#3 AND #6 AND #11 AND #16 AND #19	350

PubMed		
No.	Search Details	Results
1	"air pollution"[MeSH Terms] OR "particulate matter"[MeSH Terms]	97,797
2	"air pollut*"[Title/Abstract] OR "air contamination*"[Title/Abstract] OR "particulate matter*"[Title/Abstract] OR "PM10"[Title/Abstract] OR "PM2.5"[Title/Abstract] OR "particle*"[Title/Abstract] OR "particulat*"[Title/Abstract] OR "PM1"[Title/Abstract] OR (("precis med"[Journal] OR "phys med"[Journal] OR "pharmacogn mag"[Journal] OR "pediatr med"[Journal] OR "pm"[All Fields]) AND 1[UID])	398,421
3	"air pollution"[MeSH Terms] OR "particulate matter"[MeSH Terms] OR ("air pollut*"[Title/Abstract] OR "air contamination*"[Title/Abstract] OR "particulate matter*"[Title/Abstract] OR "PM10"[Title/Abstract] OR "PM2.5"[Title/Abstract] OR "particle*"[Title/Abstract] OR "particulat*"[Title/Abstract] OR "PM1"[Title/Abstract] OR (("precis med"[Journal] OR "phys med"[Journal] OR "pharmacogn mag"[Journal] OR "pediatr med"[Journal] OR "pm"[All Fields]) AND 1[UID]))	459,412
4	"morbidity"[MeSH Terms] OR "hospitalization"[MeSH Terms] OR "death"[MeSH Terms] OR "mortality"[MeSH Terms] OR "outpatients"[MeSH Terms]	1,255,126
5	"morbidit*"[Title/Abstract] OR "hospitalisation*"[Title/Abstract] OR "hospitalization*"[Title/Abstract] OR "death*"[Title/Abstract] OR "mortalit*"[Title/Abstract] OR "outpatien*"[Title/Abstract] OR "emergency room*"[Title/Abstract] OR "emergency department*"[Title/Abstract] OR "emergency admi*"[Title/Abstract] OR "hospital admission*"[Title/Abstract]	1,973,910
6	"morbidity"[MeSH Terms] OR "hospitalization"[MeSH Terms] OR "death"[MeSH Terms] OR "mortality"[MeSH Terms] OR "outpatients"[MeSH Terms] OR "morbidit*"[Title/Abstract] OR "hospitalisation*"[Title/Abstract] OR "hospitalization*"[Title/Abstract] OR "death*"[Title/Abstract] OR "mortalit*"[Title/Abstract] OR "outpatien*"[Title/Abstract] OR "emergency room*"[Title/Abstract] OR "emergency department*"[Title/Abstract] OR "emergency admi*"[Title/Abstract] OR "hospital admission*"[Title/Abstract]	2,772,158
7	"case-control"[Title/Abstract]	128,918

8	"cohort"[Title/Abstract] OR "follow up"[Title/Abstract] OR "Longitudinal"[Title/Abstract] OR "Prospective"[Title/Abstract] OR "Retrospective"[Title/Abstract]	2,298,002
9	"case control studies"[MeSH Terms]	1,111,995
10	"cohort studies"[MeSH Terms]	2,044,298
11	"case-control"[Title/Abstract] OR "cohort"[Title/Abstract] OR "follow up"[Title/Abstract] OR "Longitudinal"[Title/Abstract] OR "Prospective"[Title/Abstract] OR "Retrospective"[Title/Abstract] OR "case control studies"[MeSH Terms] OR "cohort studies"[MeSH Terms]	3,343,048
12	"respiratory tract diseases"[MeSH Terms]	1,378,229
13	"respirat*"[Title/Abstract] OR "pulmonary disease*"[Title/Abstract] OR "lung"[Title/Abstract] OR "chest infection*"[Title/Abstract] OR "airway"[Title/Abstract] OR "asthma*"[Title/Abstract] OR "pneumonia*"[Title/Abstract] OR "chronic obstructive pulmonary disease"[Title/Abstract] OR "COPD"[Title/Abstract]	1,391,444
14	"cardiovascular diseases"[MeSH Terms]	2,403,896
15	"cardio*"[Title/Abstract] OR "cardiop*"[Title/Abstract] OR "cardior*"[Title/Abstract] OR "heart"[Title/Abstract] OR "coronary"[Title/Abstract] OR "vascular"[Title/Abstract] OR "blood"[Title/Abstract] OR "cardiac"[Title/Abstract]	3,829,209
16	"respiratory tract diseases"[MeSH Terms] OR "respirat*"[Title/Abstract] OR "pulmonary disease*"[Title/Abstract] OR "lung"[Title/Abstract] OR "chest infection*"[Title/Abstract] OR "airway"[Title/Abstract] OR "asthma*"[Title/Abstract] OR "pneumonia*"[Title/Abstract] OR "chronic obstructive pulmonary disease"[Title/Abstract] OR "COPD"[Title/Abstract] OR "cardiovascular diseases"[MeSH Terms] OR "cardio*"[Title/Abstract] OR "cardiop*"[Title/Abstract] OR "cardior*"[Title/Abstract] OR "heart"[Title/Abstract] OR "coronary"[Title/Abstract] OR "vascular"[Title/Abstract] OR "blood"[Title/Abstract] OR "cardiac"[Title/Abstract]	6,584,293
17	"developing countries"[MeSH Terms]	75,328

18	"afghanistan"[MeSH Terms] OR "afghanistan"[All Fields] OR "afghanistan s"[All Fields] OR ("guinea"[MeSH Terms] OR "guinea"[All Fields] OR "guinea s"[All Fields] OR "guineas"[All Fields]) OR ("rwanda"[MeSH Terms] OR "rwanda"[All Fields] OR "rwanda s"[All Fields]) OR ("benin"[MeSH Terms] OR "benin"[All Fields] OR "benin s"[All Fields]) OR ("guinea bissau"[MeSH Terms] OR "guinea bissau"[All Fields] OR ("guinea"[All Fields] AND "bissau"[All Fields]) OR "guinea bissau"[All Fields]) OR ("senegal"[MeSH Terms] OR "senegal"[All Fields] OR "senegal s"[All Fields]) OR ("benin"[MeSH Terms] OR "benin"[All Fields] OR "benin s"[All Fields]) OR ("guinea bissau"[MeSH Terms] OR "guinea bissau"[All Fields] OR ("guinea"[All Fields] AND "bissau"[All Fields]) OR "guinea bissau"[All Fields]) OR ("senegal"[MeSH Terms] OR "senegal"[All Fields] OR "senegal s"[All Fields]) OR ("burkina faso"[MeSH Terms] OR ("burkina"[All Fields] AND "faso"[All Fields]) OR "burkina faso"[All Fields]) OR ("haiti"[MeSH Terms] OR "haiti"[All Fields] OR "haiti s"[All Fields]) OR ("sierra leone"[MeSH Terms] OR ("sierra"[All Fields] AND "leone"[All Fields]) OR "sierra leone"[All Fields]) OR ("burundi"[MeSH Terms] OR "burundi"[All Fields]) OR (("korea"[MeSH Terms] OR "korea"[All Fields] OR "korea s"[All Fields] OR "koreas"[All Fields]) AND "dem"[All Fields] AND ("people s"[All Fields] OR "peopled"[All Fields] OR "peopling"[All Fields] OR "persons"[MeSH Terms] OR "persons"[All Fields] OR "people"[All Fields] OR "peoples"[All Fields]) AND "Rep"[All Fields]) OR ("somalia"[MeSH Terms] OR "somalia"[All Fields]) OR ("central african republic"[MeSH Terms] OR ("central"[All Fields] AND "african"[All Fields] AND "republic"[All Fields]) OR "central african republic"[All Fields]) OR ("liberia"[MeSH Terms] OR "liberia"[All Fields] OR "liberia s"[All Fields]) OR ("south sudan"[MeSH Terms] OR ("south"[All Fields] AND "sudan"[All Fields]) OR "south sudan"[All Fields]) OR ("chad"[MeSH Terms] OR "chad"[All Fields]) OR ("madagascar"[MeSH Terms] OR "madagascar"[All Fields] OR "madagascar s"[All Fields]) OR ("tanzania"[MeSH Terms] OR "tanzania"[All Fields] OR "tanzania s"[All Fields]) OR ("comoros"[MeSH Terms] OR "comoros"[All Fields] OR "comoro"[All Fields]) OR ("malawi"[MeSH Terms] OR "malawi"[All Fields] OR "malawi s"[All Fields]) OR ("togo"[MeSH Terms] OR "togo"[All Fields]) OR (("congo"[MeSH Terms] OR "congo"[All Fields]) AND "dem"[All Fields] AND "Rep"[All Fields]) OR ("mali"[MeSH Terms] OR "mali"[All Fields]) OR ("uganda"[MeSH Terms] OR "uganda"[All Fields] OR "uganda s"[All Fields]) OR ("eritrea"[MeSH Terms] OR "eritrea"[All Fields]) OR ("mozambique"[MeSH Terms] OR "mozambique"[All Fields] OR "mozambique s"[All Fields]) OR ("zimbabwe"[MeSH Terms] OR "zimbabwe"[All Fields] OR "zimbabwe s"[All Fields]) OR	5,781,193

	("ethiopia"[MeSH Terms] OR "ethiopia"[All Fields] OR "ethiopia s"[All Fields]) OR ("nepal"[MeSH Terms] OR "nepal"[All Fields] OR "nepal s"[All Fields]) OR ("gambia"[MeSH Terms] OR "gambia"[All Fields] OR "gambia s"[All Fields]) OR ("niger"[MeSH Terms] OR "niger"[All Fields]) OR ("armenia"[MeSH Terms] OR "armenia"[All Fields]) OR ("micronesia"[MeSH Terms] OR "micronesia"[All Fields] OR "kiribati"[All Fields]) OR ("Soloman"[All Fields] AND ("island s"[All Fields] OR "islands"[MeSH Terms] OR "islands"[All Fields] OR "island"[All Fields])) OR ("bangladesh"[MeSH Terms] OR "bangladesh"[All Fields] OR "bangladesh s"[All Fields]) OR ("kosovo"[MeSH Terms] OR "kosovo"[All Fields] OR "kosovo s"[All Fields]) OR ("sri lanka"[MeSH Terms] OR ("sri"[All Fields] AND "lanka"[All Fields]) OR "sri lanka"[All Fields]) OR ("bhutan"[MeSH Terms] OR "bhutan"[All Fields] OR "bhutan s"[All Fields]) OR "kyrgyz*"[All Fields] OR ("sudan"[MeSH Terms] OR "sudan"[All Fields] OR "sudans"[All Fields] OR "sudan s"[All Fields]) OR ("bolivia"[MeSH Terms] OR "bolivia"[All Fields]) OR "Lao"[All Fields] OR ("eswatini"[MeSH Terms] OR "eswatini"[All Fields] OR "swaziland"[All Fields]) OR ("cabo verde"[MeSH Terms] OR ("cabo"[All Fields] AND "verde"[All Fields]) OR "cabo verde"[All Fields]) OR ("lesotho"[MeSH Terms] OR "lesotho"[All Fields]) OR "syria*"[All Fields] OR ("cambodia"[MeSH Terms] OR "cambodia"[All Fields] OR "cambodia s"[All Fields]) OR ("mauritania"[MeSH Terms] OR "mauritania"[All Fields]) OR ("tajikistan"[MeSH Terms] OR "tajikistan"[All Fields]) OR ("cameroon"[MeSH Terms] OR "cameroon"[All Fields] OR "cameroons"[All Fields] OR "cameroon s"[All Fields]) OR ("micronesia"[MeSH Terms] OR "micronesia"[All Fields]) OR ("timor leste"[MeSH Terms] OR "timor leste"[All Fields] OR ("timor"[All Fields] AND "leste"[All Fields]) OR "timor leste"[All Fields]) OR (("congo"[MeSH Terms] OR "congo"[All Fields]) AND "Rep"[All Fields]) OR ("moldova"[MeSH Terms] OR "moldova"[All Fields]) OR ("tonga"[MeSH Terms] OR "tonga"[All Fields] OR "tonga s"[All Fields]) OR ("cote d ivoire"[MeSH Terms] OR ("cote"[All Fields] AND "d ivoire"[All Fields]) OR "cote d ivoire"[All Fields]) OR ("mongolia"[MeSH Terms] OR "mongolia"[All Fields] OR "mongolia s"[All Fields]) OR ("tunisia"[MeSH Terms] OR "tunisia"[All Fields]) OR "Dijibouti"[All Fields] OR ("morocco"[MeSH Terms] OR "morocco"[All Fields]) OR ("ukraine"[MeSH Terms] OR "ukraine"[All Fields] OR "ukraine s"[All Fields]) OR (("egypt"[MeSH Terms] OR "egypt"[All Fields] OR "egypt s"[All Fields]) AND ("arabs"[MeSH Terms] OR "arabs"[All Fields] OR "arab"[All Fields]) AND "Rep"[All Fields]) OR ("myanmar"[MeSH Terms] OR "myanmar"[All Fields] OR "myanmars"[All Fields]) OR ("uzbekistan"[MeSH Terms] OR "uzbekistan"[All Fields]) OR ("el salvador"[MeSH Terms] OR ("el"[All	

	Fields] AND "salvador"[All Fields]) OR "el salvador"[All Fields]) OR ("nicaragua"[MeSH Terms] OR "nicaragua"[All Fields] OR "nicaragua s"[All Fields]) OR ("vanuatu"[MeSH Terms] OR "vanuatu"[All Fields]) OR ("ghana"[MeSH Terms] OR "ghana"[All Fields] OR "ghana s"[All Fields]) OR ("nigeria"[MeSH Terms] OR "nigeria"[All Fields] OR "nigeria s"[All Fields]) OR ("vietnam"[MeSH Terms] OR "vietnam"[All Fields] OR "vietnam s"[All Fields]) OR ("guatemala"[MeSH Terms] OR "guatemala"[All Fields] OR "guatemala s"[All Fields]) OR ("pakistan"[MeSH Terms] OR "pakistan"[All Fields] OR "pakistan s"[All Fields]) OR (("middle east"[MeSH Terms] OR ("middle"[All Fields] AND "east"[All Fields]) OR "middle east"[All Fields] OR ("west"[All Fields] AND "bank"[All Fields]) OR "west bank"[All Fields]) AND "Gaza"[All Fields]) OR ("honduras"[MeSH Terms] OR "honduras"[All Fields]) OR ("papua new guinea"[MeSH Terms] OR ("papua"[All Fields] AND "new"[All Fields] AND "guinea"[All Fields]) OR "papua new guinea"[All Fields]) OR "yemen*"[All Fields] OR ("india"[MeSH Terms] OR "india"[All Fields] OR "india s"[All Fields] OR "indias"[All Fields]) OR ("philippine"[All Fields] OR "philippines"[MeSH Terms] OR "philippines"[All Fields]) OR ("zambia"[MeSH Terms] OR "zambia"[All Fields] OR "zambia s"[All Fields]) OR ("indonesia"[MeSH Terms] OR "indonesia"[All Fields] OR "indonesia s"[All Fields] OR "indonesias"[All Fields]) OR ("samoa"[MeSH Terms] OR "samoa"[All Fields] OR "samoas"[All Fields]) OR ("kenya"[MeSH Terms] OR "kenya"[All Fields] OR "kenya s"[All Fields]) OR ("sao tome and principe"[MeSH Terms] OR ("sao"[All Fields] AND "tome"[All Fields] AND "principe"[All Fields]) OR "sao tome and principe"[All Fields]) OR ("albania"[MeSH Terms] OR "albania"[All Fields]) OR ("ecuador"[MeSH Terms] OR "ecuador"[All Fields] OR "ecuador s"[All Fields]) OR ("montenegro"[MeSH Terms] OR "montenegro"[All Fields]) OR ("algeria"[MeSH Terms] OR "algeria"[All Fields]) OR ("fiji"[MeSH Terms] OR "fiji"[All Fields]) OR ("namibia"[MeSH Terms] OR "namibia"[All Fields]) OR ("american samoa"[MeSH Terms] OR ("american"[All Fields] AND "samoa"[All Fields]) OR "american samoa"[All Fields]) OR ("gabon"[MeSH Terms] OR "gabon"[All Fields]) OR ("palau"[MeSH Terms] OR "palau"[All Fields] OR "palau s"[All Fields]) OR ("angola"[MeSH Terms] OR "angola"[All Fields] OR "angola s"[All Fields]) OR ("georgia"[MeSH Terms] OR "georgia"[All Fields] OR "georgia republic"[MeSH Terms] OR ("georgia"[All Fields] AND "republic"[All Fields]) OR "georgia republic"[All Fields] OR "georgia s"[All Fields]) OR ("panama"[MeSH Terms] OR "panama"[All Fields] OR "panama s"[All Fields]) OR ("argentina"[MeSH Terms] OR "argentina"[All Fields] OR "argentina s"[All Fields] OR "argentinae"[All Fields]) OR ("grenada"[MeSH Terms] OR "grenada"[All Fields]) OR ("paraguai"[All Fields] OR "paraguay"[MeSH Terms] OR	

	"paraguay"[All Fields]) OR ("azerbaijan"[MeSH Terms] OR "azerbaijan"[All Fields]) OR ("guyana"[MeSH Terms] OR "guyana"[All Fields]) OR ("peru"[MeSH Terms] OR "peru"[All Fields]) OR ("republic of belarus"[MeSH Terms] OR ("republic"[All Fields] AND "belarus"[All Fields]) OR "republic of belarus"[All Fields] OR "belarus"[All Fields]) OR (("iran"[MeSH Terms] OR "iran"[All Fields]) AND ("islam"[MeSH Terms] OR "islam"[All Fields] OR "islamic"[All Fields] OR "islam s"[All Fields] OR "islamism"[All Fields]) AND "Rep"[All Fields]) OR ("romania"[MeSH Terms] OR "romania"[All Fields] OR "romania s"[All Fields]) OR ("belize"[MeSH Terms] OR "belize"[All Fields]) OR ("iraq"[MeSH Terms] OR "iraq"[All Fields]) OR "russia*"[All Fields] OR ("bosnia and herzegovina"[MeSH Terms] OR ("bosnia"[All Fields] AND "herzegovina"[All Fields]) OR "bosnia and herzegovina"[All Fields]) OR ("jamaica"[MeSH Terms] OR "jamaica"[All Fields] OR "jamaica s"[All Fields]) OR ("serbia"[MeSH Terms] OR "serbia"[All Fields]) OR ("botswana"[MeSH Terms] OR "botswana"[All Fields] OR "botswana s"[All Fields]) OR ("jordan"[MeSH Terms] OR "jordan"[All Fields]) OR "SouthAfrica"[All Fields] OR ("brazil"[MeSH Terms] OR "brazil"[All Fields] OR "brazil s"[All Fields] OR "brazils"[All Fields]) OR ("kazakhstan"[MeSH Terms] OR "kazakhstan"[All Fields] OR "kazakhstan s"[All Fields]) OR ("saint lucia"[MeSH Terms] OR ("saint"[All Fields] AND "lucia"[All Fields]) OR "saint lucia"[All Fields] OR ("st"[All Fields] AND "lucia"[All Fields]) OR "st lucia"[All Fields]) OR ("bulgaria"[MeSH Terms] OR "bulgaria"[All Fields]) OR ("lebanon"[MeSH Terms] OR "lebanon"[All Fields] OR "lebanon s"[All Fields]) OR (vincent, st[Investigator] OR st vincent[Author] OR st vincent[Investigator]) OR ("china"[MeSH Terms] OR "china"[All Fields] OR "china s"[All Fields] OR "chinas"[All Fields]) OR ("libya"[MeSH Terms] OR "libya"[All Fields]) OR ("suriname"[MeSH Terms] OR "suriname"[All Fields] OR "surinam"[All Fields]) OR ("colombia"[MeSH Terms] OR "colombia"[All Fields] OR "colombia s"[All Fields]) OR ("republic of north macedonia"[MeSH Terms] OR ("republic"[All Fields] AND "north"[All Fields] AND "macedonia"[All Fields]) OR "republic of north macedonia"[All Fields] OR "macedonia"[All Fields]) OR ("thailand"[MeSH Terms] OR "thailand"[All Fields] OR "thailand s"[All Fields]) OR ("costa rica"[MeSH Terms] OR ("costa"[All Fields] AND "rica"[All Fields]) OR "costa rica"[All Fields]) OR ("malaysia"[MeSH Terms] OR "malaysia"[All Fields] OR "malaysia s"[All Fields]) OR ("turkey"[MeSH Terms] OR "turkey"[All Fields] OR "turkey s"[All Fields] OR "turkeys"[MeSH Terms] OR "turkeys"[All Fields]) OR ("cuba"[MeSH Terms] OR "cuba"[All Fields]) OR ("indian ocean islands"[MeSH Terms] OR ("indian"[All Fields] AND "ocean"[All Fields] AND "islands"[All Fields]) OR "indian ocean islands"[All Fields] OR "maldives"[All Fields] OR	

	"maldive"[All Fields]) OR ("turkmenistan"[MeSH Terms] OR "turkmenistan"[All Fields]) OR ("dominica"[MeSH Terms] OR "dominica"[All Fields]) OR ("micronesia"[MeSH Terms] OR "micronesia"[All Fields] OR ("marshall"[All Fields] AND "islands"[All Fields]) OR "marshall islands"[All Fields]) OR ("micronesia"[MeSH Terms] OR "micronesia"[All Fields] OR "tuvalu"[All Fields]) OR ("dominican republic"[MeSH Terms] OR ("dominican"[All Fields] AND "republic"[All Fields]) OR "dominican republic"[All Fields]) OR ("mauritius"[MeSH Terms] OR "mauritius"[All Fields]) OR ("venezuela"[MeSH Terms] OR "venezuela"[All Fields] OR "venezuela s"[All Fields]) OR ("equatorial guinea"[MeSH Terms] OR ("equatorial"[All Fields] AND "guinea"[All Fields]) OR "equatorial guinea"[All Fields]) OR ("mexico"[MeSH Terms] OR "mexico"[All Fields] OR "mexico s"[All Fields] OR "mexicos"[All Fields]) OR ("africa"[MeSH Terms] OR "africa"[All Fields] OR "africa s"[All Fields] OR "africas"[All Fields]) OR ("caribbean region"[MeSH Terms] OR ("caribbean"[All Fields] AND "region"[All Fields]) OR "caribbean region"[All Fields]) OR ("central america"[MeSH Terms] OR ("central"[All Fields] AND "america"[All Fields]) OR "central america"[All Fields]) OR "LatinAmerica"[All Fields] OR ("south america"[MeSH Terms] OR ("south"[All Fields] AND "america"[All Fields]) OR "south america"[All Fields]) OR ("asia"[MeSH Terms] OR "asia"[All Fields])	

19	"developing countries"[MeSH Terms] OR ("afghanistan"[MeSH Terms] OR "afghanistan"[All Fields] OR "afghanistan s"[All Fields] OR ("guinea"[MeSH Terms] OR "guinea"[All Fields] OR "guinea s"[All Fields] OR "guineas"[All Fields]) OR ("rwanda"[MeSH Terms] OR "rwanda"[All Fields] OR "rwanda s"[All Fields]) OR ("benin"[MeSH Terms] OR "benin"[All Fields] OR "benin s"[All Fields]) OR ("guinea bissau"[MeSH Terms] OR "guinea bissau"[All Fields] OR ("guinea"[All Fields] AND "bissau"[All Fields]) OR "guinea bissau"[All Fields]) OR ("senegal"[MeSH Terms] OR "senegal"[All Fields] OR "senegal s"[All Fields]) OR ("benin"[MeSH Terms] OR "benin"[All Fields] OR "benin s"[All Fields]) OR ("guinea bissau"[MeSH Terms] OR "guinea bissau"[All Fields] OR ("guinea"[All Fields] AND "bissau"[All Fields]) OR "guinea bissau"[All Fields]) OR ("senegal"[MeSH Terms] OR "senegal"[All Fields] OR "senegal s"[All Fields]) OR ("burkina faso"[MeSH Terms] OR ("burkina"[All Fields] AND "faso"[All Fields]) OR "burkina faso"[All Fields]) OR ("haiti"[MeSH Terms] OR "haiti"[All Fields] OR "haiti s"[All Fields]) OR ("sierra leone"[MeSH Terms] OR ("sierra"[All Fields] AND "leone"[All Fields]) OR "sierra leone"[All Fields]) OR ("burundi"[MeSH Terms] OR "burundi"[All Fields]) OR (("korea"[MeSH Terms] OR "korea"[All Fields] OR "korea s"[All Fields] OR "koreas"[All Fields]) AND "dem"[All Fields] AND ("people s"[All Fields] OR "peopled"[All Fields] OR "peopling"[All Fields] OR "persons"[MeSH Terms] OR "persons"[All Fields] OR "people"[All Fields] OR "peoples"[All Fields]) AND "Rep"[All Fields]) OR ("somalia"[MeSH Terms] OR "somalia"[All Fields]) OR ("central african republic"[MeSH Terms] OR ("central"[All Fields] AND "african"[All Fields] AND "republic"[All Fields]) OR "central african republic"[All Fields]) OR ("liberia"[MeSH Terms] OR "liberia"[All Fields] OR "liberia s"[All Fields]) OR ("south sudan"[MeSH Terms] OR ("south"[All Fields] AND "sudan"[All Fields]) OR "south sudan"[All Fields]) OR ("chad"[MeSH Terms] OR "chad"[All Fields]) OR ("madagascar"[MeSH Terms] OR "madagascar"[All Fields] OR "madagascar s"[All Fields]) OR ("tanzania"[MeSH Terms] OR "tanzania"[All Fields] OR "tanzania s"[All Fields]) OR ("comoros"[MeSH Terms] OR "comoros"[All Fields] OR "comoro"[All Fields]) OR ("malawi"[MeSH Terms] OR "malawi"[All Fields] OR "malawi s"[All Fields]) OR ("togo"[MeSH Terms] OR "togo"[All Fields]) OR (("congo"[MeSH Terms] OR "congo"[All Fields]) AND "dem"[All Fields] AND "Rep"[All Fields]) OR ("mali"[MeSH Terms] OR "mali"[All Fields]) OR ("uganda"[MeSH Terms] OR "uganda"[All Fields] OR "uganda s"[All Fields]) OR ("eritrea"[MeSH Terms] OR "eritrea"[All Fields]) OR ("mozambique"[MeSH Terms] OR "mozambique"[All Fields] OR "mozambique s"[All Fields]) OR ("zimbabwe"[MeSH Terms] OR "zimbabwe"[All Fields] OR "zimbabwe s"[All Fields]) OR	5,805,396

	("ethiopia"[MeSH Terms] OR "ethiopia"[All Fields] OR "ethiopia s"[All Fields]) OR ("nepal"[MeSH Terms] OR "nepal"[All Fields] OR "nepal s"[All Fields]) OR ("gambia"[MeSH Terms] OR "gambia"[All Fields] OR "gambia s"[All Fields]) OR ("niger"[MeSH Terms] OR "niger"[All Fields]) OR ("armenia"[MeSH Terms] OR "armenia"[All Fields]) OR ("micronesia"[MeSH Terms] OR "micronesia"[All Fields] OR "kiribati"[All Fields]) OR ("Soloman"[All Fields] AND ("island s"[All Fields] OR "islands"[MeSH Terms] OR "islands"[All Fields] OR "island"[All Fields])) OR ("bangladesh"[MeSH Terms] OR "bangladesh"[All Fields] OR "bangladesh s"[All Fields]) OR ("kosovo"[MeSH Terms] OR "kosovo"[All Fields] OR "kosovo s"[All Fields]) OR ("sri lanka"[MeSH Terms] OR ("sri"[All Fields] AND "lanka"[All Fields]) OR "sri lanka"[All Fields]) OR ("bhutan"[MeSH Terms] OR "bhutan"[All Fields] OR "bhutan s"[All Fields]) OR "kyrgyz*"[All Fields] OR ("sudan"[MeSH Terms] OR "sudan"[All Fields] OR "sudans"[All Fields] OR "sudan s"[All Fields]) OR ("bolivia"[MeSH Terms] OR "bolivia"[All Fields]) OR "Lao"[All Fields] OR ("eswatini"[MeSH Terms] OR "eswatini"[All Fields] OR "swaziland"[All Fields]) OR ("cabo verde"[MeSH Terms] OR ("cabo"[All Fields] AND "verde"[All Fields]) OR "cabo verde"[All Fields]) OR ("lesotho"[MeSH Terms] OR "lesotho"[All Fields]) OR "syria*"[All Fields] OR ("cambodia"[MeSH Terms] OR "cambodia"[All Fields] OR "cambodia s"[All Fields]) OR ("mauritania"[MeSH Terms] OR "mauritania"[All Fields]) OR ("tajikistan"[MeSH Terms] OR "tajikistan"[All Fields]) OR ("cameroon"[MeSH Terms] OR "cameroon"[All Fields] OR "cameroons"[All Fields] OR "cameroon s"[All Fields]) OR ("micronesia"[MeSH Terms] OR "micronesia"[All Fields]) OR ("timor leste"[MeSH Terms] OR "timor leste"[All Fields] OR ("timor"[All Fields] AND "leste"[All Fields]) OR "timor leste"[All Fields]) OR (("congo"[MeSH Terms] OR "congo"[All Fields]) AND "Rep"[All Fields]) OR ("moldova"[MeSH Terms] OR "moldova"[All Fields]) OR ("tonga"[MeSH Terms] OR "tonga"[All Fields] OR "tonga s"[All Fields]) OR ("cote d ivoire"[MeSH Terms] OR ("cote"[All Fields] AND "d ivoire"[All Fields]) OR "cote d ivoire"[All Fields]) OR ("mongolia"[MeSH Terms] OR "mongolia"[All Fields] OR "mongolia s"[All Fields]) OR ("tunisia"[MeSH Terms] OR "tunisia"[All Fields]) OR "Dijibouti"[All Fields] OR ("morocco"[MeSH Terms] OR "morocco"[All Fields]) OR ("ukraine"[MeSH Terms] OR "ukraine"[All Fields] OR "ukraine s"[All Fields]) OR (("egypt"[MeSH Terms] OR "egypt"[All Fields] OR "egypt s"[All Fields]) AND ("arabs"[MeSH Terms] OR "arabs"[All Fields] OR "arab"[All Fields]) AND "Rep"[All Fields]) OR ("myanmar"[MeSH Terms] OR "myanmar"[All Fields] OR "myanmars"[All Fields]) OR ("uzbekistan"[MeSH Terms] OR "uzbekistan"[All Fields]) OR ("el salvador"[MeSH Terms] OR ("el"[All	

	Fields] AND "salvador"[All Fields]) OR "el salvador"[All Fields]) OR ("nicaragua"[MeSH Terms] OR "nicaragua"[All Fields] OR "nicaragua s"[All Fields]) OR ("vanuatu"[MeSH Terms] OR "vanuatu"[All Fields]) OR ("ghana"[MeSH Terms] OR "ghana"[All Fields] OR "ghana s"[All Fields]) OR ("nigeria"[MeSH Terms] OR "nigeria"[All Fields] OR "nigeria s"[All Fields]) OR ("vietnam"[MeSH Terms] OR "vietnam"[All Fields] OR "vietnam s"[All Fields]) OR ("guatemala"[MeSH Terms] OR "guatemala"[All Fields] OR "guatemala s"[All Fields]) OR ("pakistan"[MeSH Terms] OR "pakistan"[All Fields] OR "pakistan s"[All Fields]) OR (("middle east"[MeSH Terms] OR ("middle"[All Fields] AND "east"[All Fields]) OR "middle east"[All Fields] OR ("west"[All Fields] AND "bank"[All Fields]) OR "west bank"[All Fields]) AND "Gaza"[All Fields]) OR ("honduras"[MeSH Terms] OR "honduras"[All Fields]) OR ("papua new guinea"[MeSH Terms] OR ("papua"[All Fields] AND "new"[All Fields] AND "guinea"[All Fields]) OR "papua new guinea"[All Fields]) OR "yemen*"[All Fields] OR ("india"[MeSH Terms] OR "india"[All Fields] OR "india s"[All Fields] OR "indias"[All Fields]) OR ("philippine"[All Fields] OR "philippines"[MeSH Terms] OR "philippines"[All Fields]) OR ("zambia"[MeSH Terms] OR "zambia"[All Fields] OR "zambia s"[All Fields]) OR ("indonesia"[MeSH Terms] OR "indonesia"[All Fields] OR "indonesia s"[All Fields] OR "indonesias"[All Fields]) OR ("samoa"[MeSH Terms] OR "samoa"[All Fields] OR "samoas"[All Fields]) OR ("kenya"[MeSH Terms] OR "kenya"[All Fields] OR "kenya s"[All Fields]) OR ("sao tome and principe"[MeSH Terms] OR ("sao"[All Fields] AND "tome"[All Fields] AND "principe"[All Fields]) OR "sao tome and principe"[All Fields]) OR ("albania"[MeSH Terms] OR "albania"[All Fields]) OR ("ecuador"[MeSH Terms] OR "ecuador"[All Fields] OR "ecuador s"[All Fields]) OR ("montenegro"[MeSH Terms] OR "montenegro"[All Fields]) OR ("algeria"[MeSH Terms] OR "algeria"[All Fields]) OR ("fiji"[MeSH Terms] OR "fiji"[All Fields]) OR ("namibia"[MeSH Terms] OR "namibia"[All Fields]) OR ("american samoa"[MeSH Terms] OR ("american"[All Fields] AND "samoa"[All Fields]) OR "american samoa"[All Fields]) OR ("gabon"[MeSH Terms] OR "gabon"[All Fields]) OR ("palau"[MeSH Terms] OR "palau"[All Fields] OR "palau s"[All Fields]) OR ("angola"[MeSH Terms] OR "angola"[All Fields] OR "angola s"[All Fields]) OR ("georgia"[MeSH Terms] OR "georgia"[All Fields] OR "georgia republic"[MeSH Terms] OR ("georgia"[All Fields] AND "republic"[All Fields]) OR "georgia republic"[All Fields] OR "georgia s"[All Fields]) OR ("panama"[MeSH Terms] OR "panama"[All Fields] OR "panama s"[All Fields]) OR ("argentina"[MeSH Terms] OR "argentina"[All Fields] OR "argentina s"[All Fields] OR "argentinae"[All Fields]) OR ("grenada"[MeSH Terms] OR "grenada"[All Fields]) OR ("paraguai"[All Fields] OR "paraguay"[MeSH Terms] OR	

	"paraguay"[All Fields]) OR ("azerbaijan"[MeSH Terms] OR "azerbaijan"[All Fields]) OR ("guyana"[MeSH Terms] OR "guyana"[All Fields]) OR ("peru"[MeSH Terms] OR "peru"[All Fields]) OR ("republic of belarus"[MeSH Terms] OR ("republic"[All Fields] AND "belarus"[All Fields]) OR "republic of belarus"[All Fields] OR "belarus"[All Fields]) OR (("iran"[MeSH Terms] OR "iran"[All Fields]) AND ("islam"[MeSH Terms] OR "islam"[All Fields] OR "islamic"[All Fields] OR "islam s"[All Fields] OR "islamism"[All Fields]) AND "Rep"[All Fields]) OR ("romania"[MeSH Terms] OR "romania"[All Fields] OR "romania s"[All Fields]) OR ("belize"[MeSH Terms] OR "belize"[All Fields]) OR ("iraq"[MeSH Terms] OR "iraq"[All Fields]) OR "russia*"[All Fields] OR ("bosnia and herzegovina"[MeSH Terms] OR ("bosnia"[All Fields] AND "herzegovina"[All Fields]) OR "bosnia and herzegovina"[All Fields]) OR ("jamaica"[MeSH Terms] OR "jamaica"[All Fields] OR "jamaica s"[All Fields]) OR ("serbia"[MeSH Terms] OR "serbia"[All Fields]) OR ("botswana"[MeSH Terms] OR "botswana"[All Fields] OR "botswana s"[All Fields]) OR ("jordan"[MeSH Terms] OR "jordan"[All Fields]) OR "SouthAfrica"[All Fields] OR ("brazil"[MeSH Terms] OR "brazil"[All Fields] OR "brazil s"[All Fields] OR "brazils"[All Fields]) OR ("kazakhstan"[MeSH Terms] OR "kazakhstan"[All Fields] OR "kazakhstan s"[All Fields]) OR ("saint lucia"[MeSH Terms] OR ("saint"[All Fields] AND "lucia"[All Fields]) OR "saint lucia"[All Fields] OR ("st"[All Fields] AND "lucia"[All Fields]) OR "st lucia"[All Fields]) OR ("bulgaria"[MeSH Terms] OR "bulgaria"[All Fields]) OR ("lebanon"[MeSH Terms] OR "lebanon"[All Fields] OR "lebanon s"[All Fields]) OR (vincent, st[Investigator] OR st vincent[Author] OR st vincent[Investigator]) OR ("china"[MeSH Terms] OR "china"[All Fields] OR "china s"[All Fields] OR "chinas"[All Fields]) OR ("libya"[MeSH Terms] OR "libya"[All Fields]) OR ("suriname"[MeSH Terms] OR "suriname"[All Fields] OR "surinam"[All Fields]) OR ("colombia"[MeSH Terms] OR "colombia"[All Fields] OR "colombia s"[All Fields]) OR ("republic of north macedonia"[MeSH Terms] OR ("republic"[All Fields] AND "north"[All Fields] AND "macedonia"[All Fields]) OR "republic of north macedonia"[All Fields] OR "macedonia"[All Fields]) OR ("thailand"[MeSH Terms] OR "thailand"[All Fields] OR "thailand s"[All Fields]) OR ("costa rica"[MeSH Terms] OR ("costa"[All Fields] AND "rica"[All Fields]) OR "costa rica"[All Fields]) OR ("malaysia"[MeSH Terms] OR "malaysia"[All Fields] OR "malaysia s"[All Fields]) OR ("turkey"[MeSH Terms] OR "turkey"[All Fields] OR "turkey s"[All Fields] OR "turkeys"[MeSH Terms] OR "turkeys"[All Fields]) OR ("cuba"[MeSH Terms] OR "cuba"[All Fields]) OR ("indian ocean islands"[MeSH Terms] OR ("indian"[All Fields] AND "ocean"[All Fields] AND "islands"[All Fields]) OR "indian ocean islands"[All Fields] OR "maldives"[All Fields] OR	

	"maldive"[All Fields]) OR ("turkmenistan"[MeSH Terms] OR "turkmenistan"[All Fields]) OR ("dominica"[MeSH Terms] OR "dominica"[All Fields]) OR ("micronesia"[MeSH Terms] OR "micronesia"[All Fields] OR ("marshall"[All Fields] AND "islands"[All Fields]) OR "marshall islands"[All Fields]) OR ("micronesia"[MeSH Terms] OR "micronesia"[All Fields] OR "tuvalu"[All Fields]) OR ("dominican republic"[MeSH Terms] OR ("dominican"[All Fields] AND "republic"[All Fields]) OR "dominican republic"[All Fields]) OR ("mauritius"[MeSH Terms] OR "mauritius"[All Fields]) OR ("venezuela"[MeSH Terms] OR "venezuela"[All Fields] OR "venezuela s"[All Fields]) OR ("equatorial guinea"[MeSH Terms] OR ("equatorial"[All Fields] AND "guinea"[All Fields]) OR "equatorial guinea"[All Fields]) OR ("mexico"[MeSH Terms] OR "mexico"[All Fields] OR "mexico s"[All Fields] OR "mexicos"[All Fields]) OR ("africa"[MeSH Terms] OR "africa"[All Fields] OR "africa s"[All Fields] OR "africas"[All Fields]) OR ("caribbean region"[MeSH Terms] OR ("caribbean"[All Fields] AND "region"[All Fields]) OR "caribbean region"[All Fields]) OR ("central america"[MeSH Terms] OR ("central"[All Fields] AND "america"[All Fields]) OR "central america"[All Fields]) OR "LatinAmerica"[All Fields] OR ("south america"[MeSH Terms] OR ("south"[All Fields] AND "america"[All Fields]) OR "south america"[All Fields]) OR ("asia"[MeSH Terms] OR "asia"[All Fields]))	

20	("air pollution"[MeSH Terms] OR "particulate matter"[MeSH Terms] OR ("air pollut*"[Title/Abstract] OR "air contamination*"[Title/Abstract] OR "particulate matter*"[Title/Abstract] OR "PM10"[Title/Abstract] OR "PM2.5"[Title/Abstract] OR "particle*"[Title/Abstract] OR "particulat*"[Title/Abstract] OR "PM1"[Title/Abstract] OR (("precis med"[Journal] OR "phys med"[Journal] OR "pharmacogn mag"[Journal] OR "pediatr med"[Journal] OR "pm"[All Fields]) AND 1[UID]))) AND ("morbidity"[MeSH Terms] OR "hospitalization"[MeSH Terms] OR "death"[MeSH Terms] OR "mortality"[MeSH Terms] OR "outpatients"[MeSH Terms] OR ("morbidit*"[Title/Abstract] OR "hospitalisation*"[Title/Abstract] OR "hospitalization*"[Title/Abstract] OR "death*"[Title/Abstract] OR "mortalit*"[Title/Abstract] OR "outpatien*"[Title/Abstract] OR "emergency room*"[Title/Abstract] OR "emergency department*"[Title/Abstract] OR "emergency admi*"[Title/Abstract] OR "hospital admission*"[Title/Abstract])) AND ("case-control"[Title/Abstract] OR ("cohort"[Title/Abstract] OR "follow up"[Title/Abstract] OR "Longitudinal"[Title/Abstract] OR "Prospective"[Title/Abstract] OR "Retrospective"[Title/Abstract]) OR "case control studies"[MeSH Terms] OR "cohort studies"[MeSH Terms]) AND ("respiratory tract diseases"[MeSH Terms] OR ("respirat*"[Title/Abstract] OR "pulmonary disease*"[Title/Abstract] OR "lung"[Title/Abstract] OR "chest infection*"[Title/Abstract] OR "airway"[Title/Abstract] OR "asthma*"[Title/Abstract] OR "pneumonia*"[Title/Abstract] OR "chronic obstructive pulmonary disease"[Title/Abstract] OR "COPD"[Title/Abstract]) OR "cardiovascular diseases"[MeSH Terms] OR ("cardio*"[Title/Abstract] OR "cardiop*"[Title/Abstract] OR "cardior*"[Title/Abstract] OR "heart"[Title/Abstract] OR "coronary"[Title/Abstract] OR "vascular"[Title/Abstract] OR "blood"[Title/Abstract] OR "cardiac"[Title/Abstract])) AND ("developing countries"[MeSH Terms] OR ("afghanistan"[MeSH Terms] OR "afghanistan"[All Fields] OR "afghanistan s"[All Fields] OR ("guinea"[MeSH Terms] OR "guinea"[All Fields] OR "guinea s"[All Fields] OR "guineas"[All Fields]) OR ("rwanda"[MeSH Terms] OR "rwanda"[All Fields] OR "rwanda s"[All Fields]) OR ("benin"[MeSH Terms] OR "benin"[All Fields] OR "benin s"[All Fields]) OR ("guinea bissau"[MeSH Terms] OR "guinea bissau"[All Fields] OR ("guinea"[All Fields] AND "bissau"[All Fields]) OR "guinea bissau"[All Fields]) OR ("senegal"[MeSH Terms] OR "senegal"[All Fields] OR "senegal s"[All Fields]) OR ("benin"[MeSH Terms] OR "benin"[All Fields] OR "benin s"[All Fields]) OR ("guinea bissau"[MeSH Terms] OR "guinea bissau"[All Fields] OR ("guinea"[All Fields] AND "bissau"[All Fields]) OR "guinea bissau"[All Fields]) OR ("senegal"[MeSH Terms] OR "senegal"[All Fields] OR "senegal s"[All Fields]) OR ("burkina faso"[MeSH Terms] OR	990

	("burkina"[All Fields] AND "faso"[All Fields]) OR "burkina faso"[All Fields]) OR ("haiti"[MeSH Terms] OR "haiti"[All Fields] OR "haiti s"[All Fields]) OR ("sierra leone"[MeSH Terms] OR ("sierra"[All Fields] AND "leone"[All Fields]) OR "sierra leone"[All Fields]) OR ("burundi"[MeSH Terms] OR "burundi"[All Fields]) OR (("korea"[MeSH Terms] OR "korea"[All Fields] OR "korea s"[All Fields] OR "koreas"[All Fields]) AND "dem"[All Fields] AND ("people s"[All Fields] OR "peopled"[All Fields] OR "peopling"[All Fields] OR "persons"[MeSH Terms] OR "persons"[All Fields] OR "people"[All Fields] OR "peoples"[All Fields]) AND "Rep"[All Fields]) OR ("somalia"[MeSH Terms] OR "somalia"[All Fields]) OR ("central african republic"[MeSH Terms] OR ("central"[All Fields] AND "african"[All Fields] AND "republic"[All Fields]) OR "central african republic"[All Fields]) OR ("liberia"[MeSH Terms] OR "liberia"[All Fields] OR "liberia s"[All Fields]) OR ("south sudan"[MeSH Terms] OR ("south"[All Fields] AND "sudan"[All Fields]) OR "south sudan"[All Fields]) OR ("chad"[MeSH Terms] OR "chad"[All Fields]) OR ("madagascar"[MeSH Terms] OR "madagascar"[All Fields] OR "madagascar s"[All Fields]) OR ("tanzania"[MeSH Terms] OR "tanzania"[All Fields] OR "tanzania s"[All Fields]) OR ("comoros"[MeSH Terms] OR "comoros"[All Fields] OR "comoro"[All Fields]) OR ("malawi"[MeSH Terms] OR "malawi"[All Fields] OR "malawi s"[All Fields]) OR ("togo"[MeSH Terms] OR "togo"[All Fields]) OR (("congo"[MeSH Terms] OR "congo"[All Fields]) AND "dem"[All Fields] AND "Rep"[All Fields]) OR ("mali"[MeSH Terms] OR "mali"[All Fields]) OR ("uganda"[MeSH Terms] OR "uganda"[All Fields] OR "uganda s"[All Fields]) OR ("eritrea"[MeSH Terms] OR "eritrea"[All Fields]) OR ("mozambique"[MeSH Terms] OR "mozambique"[All Fields] OR "mozambique s"[All Fields]) OR ("zimbabwe"[MeSH Terms] OR "zimbabwe"[All Fields] OR "zimbabwe s"[All Fields]) OR ("ethiopia"[MeSH Terms] OR "ethiopia"[All Fields] OR "ethiopia s"[All Fields]) OR ("nepal"[MeSH Terms] OR "nepal"[All Fields] OR "nepal s"[All Fields]) OR ("gambia"[MeSH Terms] OR "gambia"[All Fields] OR "gambia s"[All Fields]) OR ("niger"[MeSH Terms] OR "niger"[All Fields]) OR ("armenia"[MeSH Terms] OR "armenia"[All Fields]) OR ("micronesia"[MeSH Terms] OR "micronesia"[All Fields] OR "kiribati"[All Fields]) OR ("Soloman"[All Fields] AND ("island s"[All Fields] OR "islands"[MeSH Terms] OR "islands"[All Fields] OR "island"[All Fields])) OR ("bangladesh"[MeSH Terms] OR "bangladesh"[All Fields] OR "bangladesh s"[All Fields]) OR ("kosovo"[MeSH Terms] OR "kosovo"[All Fields] OR "kosovo s"[All Fields]) OR ("sri lanka"[MeSH Terms] OR ("sri"[All Fields] AND "lanka"[All Fields]) OR "sri lanka"[All Fields]) OR ("bhutan"[MeSH Terms] OR "bhutan"[All Fields] OR "bhutan s"[All Fields]) OR "kyrgyz*"[All	

	Fields] OR ("sudan"[MeSH Terms] OR "sudan"[All Fields] OR "sudans"[All Fields] OR "sudan s"[All Fields]) OR ("bolivia"[MeSH Terms] OR "bolivia"[All Fields]) OR "Lao"[All Fields] OR ("eswatini"[MeSH Terms] OR "eswatini"[All Fields] OR "swaziland"[All Fields]) OR ("cabo verde"[MeSH Terms] OR ("cabo"[All Fields] AND "verde"[All Fields]) OR "cabo verde"[All Fields]) OR ("lesotho"[MeSH Terms] OR "lesotho"[All Fields]) OR "syria*"[All Fields] OR ("cambodia"[MeSH Terms] OR "cambodia"[All Fields] OR "cambodia s"[All Fields]) OR ("mauritania"[MeSH Terms] OR "mauritania"[All Fields]) OR ("tajikistan"[MeSH Terms] OR "tajikistan"[All Fields]) OR ("cameroon"[MeSH Terms] OR "cameroon"[All Fields] OR "cameroons"[All Fields] OR "cameroon s"[All Fields]) OR ("micronesia"[MeSH Terms] OR "micronesia"[All Fields]) OR ("timor leste"[MeSH Terms] OR "timor leste"[All Fields] OR ("timor"[All Fields] AND "leste"[All Fields]) OR "timor leste"[All Fields]) OR (("congo"[MeSH Terms] OR "congo"[All Fields]) AND "Rep"[All Fields]) OR ("moldova"[MeSH Terms] OR "moldova"[All Fields]) OR ("tonga"[MeSH Terms] OR "tonga"[All Fields] OR "tonga s"[All Fields]) OR ("cote d ivoire"[MeSH Terms] OR ("cote"[All Fields] AND "d ivoire"[All Fields]) OR "cote d ivoire"[All Fields]) OR ("mongolia"[MeSH Terms] OR "mongolia"[All Fields] OR "mongolia s"[All Fields]) OR ("tunisia"[MeSH Terms] OR "tunisia"[All Fields]) OR "Dijibouti"[All Fields] OR ("morocco"[MeSH Terms] OR "morocco"[All Fields]) OR ("ukraine"[MeSH Terms] OR "ukraine"[All Fields] OR "ukraine s"[All Fields]) OR (("egypt"[MeSH Terms] OR "egypt"[All Fields] OR "egypt s"[All Fields]) AND ("arabs"[MeSH Terms] OR "arabs"[All Fields] OR "arab"[All Fields]) AND "Rep"[All Fields]) OR ("myanmar"[MeSH Terms] OR "myanmar"[All Fields] OR "myanmars"[All Fields]) OR ("uzbekistan"[MeSH Terms] OR "uzbekistan"[All Fields]) OR ("el salvador"[MeSH Terms] OR ("el"[All Fields] AND "salvador"[All Fields]) OR "el salvador"[All Fields]) OR ("nicaragua"[MeSH Terms] OR "nicaragua"[All Fields] OR "nicaragua s"[All Fields]) OR ("vanuatu"[MeSH Terms] OR "vanuatu"[All Fields]) OR ("ghana"[MeSH Terms] OR "ghana"[All Fields] OR "ghana s"[All Fields]) OR ("nigeria"[MeSH Terms] OR "nigeria"[All Fields] OR "nigeria s"[All Fields]) OR ("vietnam"[MeSH Terms] OR "vietnam"[All Fields] OR "vietnam s"[All Fields]) OR ("guatemala"[MeSH Terms] OR "guatemala"[All Fields] OR "guatemala s"[All Fields]) OR ("pakistan"[MeSH Terms] OR "pakistan"[All Fields] OR "pakistan s"[All Fields]) OR (("middle east"[MeSH Terms] OR ("middle"[All Fields] AND "east"[All Fields]) OR "middle east"[All Fields] OR ("west"[All Fields] AND "bank"[All Fields]) OR "west bank"[All Fields]) AND "Gaza"[All Fields]) OR ("honduras"[MeSH Terms] OR "honduras"[All Fields]) OR ("papua new guinea"[MeSH Terms] OR ("papua"[All	

	Fields] AND "new"[All Fields] AND "guinea"[All Fields]) OR "papua new guinea"[All Fields]) OR "yemen*"[All Fields] OR ("india"[MeSH Terms] OR "india"[All Fields] OR "india s"[All Fields] OR "indias"[All Fields]) OR ("philippine"[All Fields] OR "philippines"[MeSH Terms] OR "philippines"[All Fields]) OR ("zambia"[MeSH Terms] OR "zambia"[All Fields] OR "zambia s"[All Fields]) OR ("indonesia"[MeSH Terms] OR "indonesia"[All Fields] OR "indonesia s"[All Fields] OR "indonesias"[All Fields]) OR ("samoa"[MeSH Terms] OR "samoa"[All Fields] OR "samoas"[All Fields]) OR ("kenya"[MeSH Terms] OR "kenya"[All Fields] OR "kenya s"[All Fields]) OR ("sao tome and principe"[MeSH Terms] OR ("sao"[All Fields] AND "tome"[All Fields] AND "principe"[All Fields]) OR "sao tome and principe"[All Fields]) OR ("albania"[MeSH Terms] OR "albania"[All Fields]) OR ("ecuador"[MeSH Terms] OR "ecuador"[All Fields] OR "ecuador s"[All Fields]) OR ("montenegro"[MeSH Terms] OR "montenegro"[All Fields]) OR ("algeria"[MeSH Terms] OR "algeria"[All Fields]) OR ("fiji"[MeSH Terms] OR "fiji"[All Fields]) OR ("namibia"[MeSH Terms] OR "namibia"[All Fields]) OR ("american samoa"[MeSH Terms] OR ("american"[All Fields] AND "samoa"[All Fields]) OR "american samoa"[All Fields]) OR ("gabon"[MeSH Terms] OR "gabon"[All Fields]) OR ("palau"[MeSH Terms] OR "palau"[All Fields] OR "palau s"[All Fields]) OR ("angola"[MeSH Terms] OR "angola"[All Fields] OR "angola s"[All Fields]) OR ("georgia"[MeSH Terms] OR "georgia"[All Fields] OR "georgia republic"[MeSH Terms] OR ("georgia"[All Fields] AND "republic"[All Fields]) OR "georgia republic"[All Fields] OR "georgia s"[All Fields]) OR ("panama"[MeSH Terms] OR "panama"[All Fields] OR "panama s"[All Fields]) OR ("argentina"[MeSH Terms] OR "argentina"[All Fields] OR "argentina s"[All Fields] OR "argentinae"[All Fields]) OR ("grenada"[MeSH Terms] OR "grenada"[All Fields]) OR ("paraguai"[All Fields] OR "paraguay"[MeSH Terms] OR "paraguay"[All Fields]) OR ("azerbaijan"[MeSH Terms] OR "azerbaijan"[All Fields]) OR ("guyana"[MeSH Terms] OR "guyana"[All Fields]) OR ("peru"[MeSH Terms] OR "peru"[All Fields]) OR ("republic of belarus"[MeSH Terms] OR ("republic"[All Fields] AND "belarus"[All Fields]) OR "republic of belarus"[All Fields] OR "belarus"[All Fields]) OR (("iran"[MeSH Terms] OR "iran"[All Fields]) AND ("islam"[MeSH Terms] OR "islam"[All Fields] OR "islamic"[All Fields] OR "islam s"[All Fields] OR "islamism"[All Fields]) AND "Rep"[All Fields]) OR ("romania"[MeSH Terms] OR "romania"[All Fields] OR "romania s"[All Fields]) OR ("belize"[MeSH Terms] OR "belize"[All Fields]) OR ("iraq"[MeSH Terms] OR "iraq"[All Fields]) OR "russia*"[All Fields] OR ("bosnia and herzegovina"[MeSH Terms] OR ("bosnia"[All Fields] AND "herzegovina"[All Fields]) OR "bosnia and herzegovina"[All Fields]) OR ("jamaica"[MeSH Terms] OR		

	"jamaica"[All Fields] OR "jamaica s"[All Fields]) OR ("serbia"[MeSH Terms] OR "serbia"[All Fields]) OR ("botswana"[MeSH Terms] OR "botswana"[All Fields] OR "botswana s"[All Fields]) OR ("jordan"[MeSH Terms] OR "jordan"[All Fields]) OR "SouthAfrica"[All Fields] OR ("brazil"[MeSH Terms] OR "brazil"[All Fields] OR "brazil s"[All Fields] OR "brazils"[All Fields]) OR ("kazakhstan"[MeSH Terms] OR "kazakhstan"[All Fields] OR "kazakhstan s"[All Fields]) OR ("saint lucia"[MeSH Terms] OR ("saint"[All Fields] AND "lucia"[All Fields]) OR "saint lucia"[All Fields] OR ("st"[All Fields] AND "lucia"[All Fields]) OR "st lucia"[All Fields]) OR ("bulgaria"[MeSH Terms] OR "bulgaria"[All Fields]) OR ("lebanon"[MeSH Terms] OR "lebanon"[All Fields] OR "lebanon s"[All Fields]) OR (vincent, st[Investigator] OR st vincent[Author] OR st vincent[Investigator]) OR ("china"[MeSH Terms] OR "china"[All Fields] OR "china s"[All Fields] OR "chinas"[All Fields]) OR ("libya"[MeSH Terms] OR "libya"[All Fields]) OR ("suriname"[MeSH Terms] OR "suriname"[All Fields] OR "surinam"[All Fields]) OR ("colombia"[MeSH Terms] OR "colombia"[All Fields] OR "colombia s"[All Fields]) OR ("republic of north macedonia"[MeSH Terms] OR ("republic"[All Fields] AND "north"[All Fields] AND "macedonia"[All Fields]) OR "republic of north macedonia"[All Fields] OR "macedonia"[All Fields]) OR ("thailand"[MeSH Terms] OR "thailand"[All Fields] OR "thailand s"[All Fields]) OR ("costa rica"[MeSH Terms] OR ("costa"[All Fields] AND "rica"[All Fields]) OR "costa rica"[All Fields]) OR ("malaysia"[MeSH Terms] OR "malaysia"[All Fields] OR "malaysia s"[All Fields]) OR ("turkey"[MeSH Terms] OR "turkey"[All Fields] OR "turkey s"[All Fields] OR "turkeys"[MeSH Terms] OR "turkeys"[All Fields]) OR ("cuba"[MeSH Terms] OR "cuba"[All Fields]) OR ("indian ocean islands"[MeSH Terms] OR ("indian"[All Fields] AND "ocean"[All Fields] AND "islands"[All Fields]) OR "indian ocean islands"[All Fields] OR "maldives"[All Fields] OR "maldive"[All Fields]) OR ("turkmenistan"[MeSH Terms] OR "turkmenistan"[All Fields]) OR ("dominica"[MeSH Terms] OR "dominica"[All Fields]) OR ("micronesia"[MeSH Terms] OR "micronesia"[All Fields] OR ("marshall"[All Fields] AND "islands"[All Fields]) OR "marshall islands"[All Fields]) OR ("micronesia"[MeSH Terms] OR "micronesia"[All Fields] OR "tuvalu"[All Fields]) OR ("dominican republic"[MeSH Terms] OR ("dominican"[All Fields] AND "republic"[All Fields]) OR "dominican republic"[All Fields]) OR ("mauritius"[MeSH Terms] OR "mauritius"[All Fields]) OR ("venezuela"[MeSH Terms] OR "venezuela"[All Fields] OR "venezuela s"[All Fields]) OR ("equatorial guinea"[MeSH Terms] OR ("equatorial"[All Fields] AND "guinea"[All Fields]) OR "equatorial guinea"[All Fields]) OR ("mexico"[MeSH Terms] OR "mexico"[All Fields] OR "mexico s"[All Fields] OR "mexicos"[All Fields])	

	OR ("africa"[MeSH Terms] OR "africa"[All Fields] OR "africa s"[All Fields] OR "africas"[All Fields]) OR ("caribbean region"[MeSH Terms] OR ("caribbean"[All Fields] AND "region"[All Fields]) OR "caribbean region"[All Fields]) OR ("central america"[MeSH Terms] OR ("central"[All Fields] AND "america"[All Fields]) OR "central america"[All Fields]) OR "LatinAmerica"[All Fields] OR ("south america"[MeSH Terms] OR ("south"[All Fields] AND "america"[All Fields]) OR "south america"[All Fields]) OR ("asia"[MeSH Terms] OR "asia"[All Fields])))	

Web of Science		
No.	Search Details	Results
1	TS=(Air Pollution) OR TS= (Particulate Matter)	136,609
2	TS=(air pollut*) OR TS=(air contamination*) OR TS=(particulate matter*) OR TS=(PM 10) OR TS=(PM2.5) OR TS=(particle*) OR TS=(particulat*) OR TS=(PM1) OR TS=(PM(1))	1,648,088
3	#2 OR #1	1,648,088
4	TS=(morbidity) OR TS=(hospitalization) ORTS= (death) OR TS=(mortality) OR TS=(outpatient)	1,995,704
5	TS=(morbidity) OR TS=(hospitalisation*) ORTS=(hospitalization*) OR TS=(death,) OR TS=(mortalit*) OR TS=(outpatient) OR TS= (emergency room*) OR TS=(emergency department*) OR TS=(emergency admf) OR TS=(hospital admission*)	2,120,414
6	#5 OR #4	2,120,414
7	TS=(case-control)	125,809
8	TS=(cohort) OR TS=(follow up) OR TS=(Longitudinal) OR TS=(Prospective) OR TS=(Retrospective)	2,732,654
9	TS=(Case-Control Studies)	121,302
10	TS=(Cohort Studies)	518,571
11	#10 OR #9 OR #8 OR #7	2,814,493
12	TS=(respiratory tract disease)	22,961
13	TS=(respirat*) OR TS= (pulmonary disease*) OR TS=(lung) OR TS= (chest infection*) OR TS=(airway) OR TS=(asthma*) OR TS= (pneumonia*) OR TS=(chronic obstructive pulmonary disease) OR TS= (COPD)	1,595,859
14	TS=(cardiovascular diseases)	343,460
15	TS=(cardio*) OR TS=(cardiop*) ORTS=(cardloL) ORTS=(heart) ORTS= (coronary) ORTS=(vascular) ORTS=(blood) OR TS=(cardiac)	3,820,202
16	#15 OR #14 OR #13 OR #12	5,087,796
17	TS=(developing country)	234,815

18	afghanistanTS= OR afghanistanTS= OR afghanistansTS= OR (guineaTS= OR guineaTS= OR guinea sTS= OR guineasTS=) OR (rwandaTS= OR rwandaTS= OR rwanda sTS=) OR (beninTS= OR beninTS= OR benin sTS=) OR (guinea bissauTS= OR guinea bissauTS= OR (guineaTS= AND bissauTS=) OR guinea bissauTS=) OR (senegalTS= OR senegalTS= OR senegal sTS=) OR (beninTS= OR beninTS= OR benin sTS=) OR (guinea bissauTS= OR guinea bissauTS= OR (guineaTS= AND bissauTS=) OR guinea bissauTS=) OR (senegalTS= OR senegalTS= OR senegal sTS=) OR (burkina fasoTS= OR (burkinaTS= AND fasoTS=) OR burkina fasoTS=) OR (haitiTS= OR haitiTS= OR haiti sTS=) OR (sierra leoneTS= OR (sierraTS= AND leoneTS=) OR sierra leoneTS=) OR (burundiTS= OR burundiTS=) OR ((koreaTS= OR koreaTS= OR korea sTS= OR koreasTS=) AND demTS= AND (people sTS= OR peopledTS= OR peoplingTS= OR personsTS= OR personsTS= OR peopleTS= OR peoplesTS=) AND RepTS=) OR (somaliaTS= OR somaliaTS=) OR (central african republicTS= OR (centralTS= AND africanTS= AND republicTS=) OR central african republicTS=) OR (liberiaTS= OR liberiaTS= OR liberia sTS=) OR (south sudanTS= OR (southTS= AND sudanTS=) OR south sudanTS=) OR (chadTS= OR chadTS=) OR (madagascarTS= OR madagascarTS= OR madagascar sTS=) OR (tanzaniaTS= OR tanzaniaTS= OR tanzania sTS=) OR (comorosTS= OR comorosTS= OR comoroTS=) OR (malawiTS= OR malawiTS= OR malawi sTS=) OR (togoTS= OR togoTS=) OR ((congoTS= OR congoTS=) AND demTS= AND RepTS=) OR (maliTS= OR maliTS=) OR (ugandaTS= OR ugandaTS= OR uganda sTS=) OR (eritreaTS= OR eritreaTS=) OR (mozambiqueTS= OR mozambiqueTS= OR mozambique sTS=) OR (zimbabweTS= OR zimbabweTS= OR zimbabwe sTS=) OR (ethiopiaTS= OR ethiopiaTS= OR ethiopia sTS=) OR (nepalTS= OR nepalTS= OR nepal sTS=) OR (gambiaTS= OR gambiaTS= OR gambia sTS=) OR (nigerTS= OR nigerTS=) OR (armeniaTS= OR armeniaTS=) OR (micronesiaTS= OR micronesiaTS= OR kiribatiTS=) OR (SolomanTS= AND (island sTS= OR islandsTS= OR islandsTS= OR islandTS=)) OR (bangladeshTS= OR bangladeshTS= OR bangladesh sTS=) OR (kosovoTS= OR kosovoTS= OR kosovo sTS=) OR (sri lankaTS= OR (sriTS= AND lankaTS=) OR sri lankaTS=) OR (bhutanTS= OR bhutanTS= OR bhutan sTS=) OR kyrgyz*TS= OR (sudanTS= OR sudanTS= OR sudansTS= OR sudan sTS=) OR (boliviaTS= OR boliviaTS=) OR LaoTS= OR (eswatiniTS= OR eswatiniTS= OR swazilandTS=) OR (cabo verdeTS= OR (caboTS= AND verdeTS=) OR cabo verdeTS=) OR (lesothoTS= OR lesothoTS=) OR syria*TS= OR (cambodiaTS= OR cambodiaTS= OR cambodia sTS=) OR (mauritaniaTS= OR mauritaniaTS=) OR (tajikistanTS= OR tajikistanTS=) OR (cameroonTS= OR cameroonTS=	2,648,354

	OR cameroonsTS= OR cameroon sTS=) OR (micronesiaTS= OR micronesiaTS=) OR (timor lesteTS= OR timor lesteTS= OR (timorTS= AND lesteTS=) OR timor lesteTS=) OR ((congoTS= OR congoTS=) AND RepTS=) OR (moldovaTS= OR moldovaTS=) OR (tongaTS= OR tongaTS= OR tonga sTS=) OR (cote d ivoireTS= OR (coteTS= AND d ivoireTS=) OR cote d ivoireTS=) OR (mongoliaTS= OR mongoliaTS= OR mongolia sTS=) OR (tunisiaTS= OR tunisiaTS=) OR DijiboutiTS= OR (moroccoTS= OR moroccoTS=) OR (ukraineTS= OR ukraineTS= OR ukraine sTS=) OR ((egyptTS= OR egyptTS= OR egypt sTS=) AND (arabsTS= OR arabsTS= OR arabTS=) AND RepTS=) OR (myanmarTS= OR myanmarTS= OR myanmarsTS=) OR (uzbekistanTS= OR uzbekistanTS=) OR (el salvadorTS= OR (elTS= AND salvadorTS=) OR el salvadorTS=) OR (nicaraguaTS= OR nicaraguaTS= OR nicaragua sTS=) OR (vanuatuTS= OR vanuatuTS=) OR (ghanaTS= OR ghanaTS= OR ghana sTS=) OR (nigeriaTS= OR nigeriaTS= OR nigeria sTS=) OR (vietnamTS= OR vietnamTS= OR vietnam sTS=) OR (guatemalaTS= OR guatemalaTS= OR guatemala sTS=) OR (pakistanTS= OR pakistanTS= OR pakistan sTS=) OR ((middle eastTS= OR (middleTS= AND eastTS=) OR middle eastTS= OR (westTS= AND bankTS=) OR west bankTS=) AND GazaTS=) OR (hondurasTS= OR hondurasTS=) OR (papua new guineaTS= OR (papuaTS= AND newTS= AND guineaTS=) OR papua new guineaTS=) OR yemen*TS= OR (indiaTS= OR indiaTS= OR india sTS= OR indiasTS=) OR (philippineTS= OR philippinesTS= OR philippinesTS=) OR (zambiaTS= OR zambiaTS= OR zambia sTS=) OR (indonesiaTS= OR indonesiaTS= OR indonesia sTS= OR indonesiasTS=) OR (samoaTS= OR samoaTS= OR samoasTS=) OR (kenyaTS= OR kenyaTS= OR kenya sTS=) OR (sao tome and principeTS= OR (saoTS= AND tomeTS= AND principeTS=) OR sao tome and principeTS=) OR (albaniaTS= OR albaniaTS=) OR (ecuadorTS= OR ecuadorTS= OR ecuador sTS=) OR (montenegroTS= OR montenegroTS=) OR (algeriaTS= OR algeriaTS=) OR (fijiTS= OR fijiTS=) OR (namibiaTS= OR namibiaTS=) OR (american samoaTS= OR (americanTS= AND samoaTS=) OR american samoaTS=) OR (gabonTS= OR gabonTS=) OR (palauTS= OR palauTS= OR palau sTS=) OR (angolaTS= OR angolaTS= OR angola sTS=) OR (georgiaTS= OR georgiaTS= OR georgia republicTS= OR (georgiaTS= AND republicTS=) OR georgia republicTS= OR georgia sTS=) OR (panamaTS= OR panamaTS= OR panama sTS=) OR (argentinaTS= OR argentinaTS= OR argentina sTS= OR argentinaeTS=) OR (grenadaTS= OR grenadaTS=) OR (paraguaiTS= OR paraguayTS= OR paraguayTS=) OR (azerbaijanTS= OR azerbaijanTS=) OR (guyanaTS= OR guyanaTS=) OR (peruTS= OR peruTS=) OR (republic of belarusTS= OR (republicTS= AND belarusTS=) OR republic of belarusTS= OR belarusTS=) OR	

		((iranTS= OR iranTS=) AND (islamTS= OR islamTS= OR islamicTS= OR islam sTS= OR islamismTS=) AND RepTS=) OR (romaniaTS= OR romaniaTS= OR romania sTS=) OR (belizeTS= OR belizeTS=) OR (iraqTS= OR iraqTS=) OR russia*TS= OR (bosnia and herzegovinaTS= OR (bosniaTS= AND herzegovinaTS=) OR bosnia and herzegovinaTS=) OR (jamaicaTS= OR jamaicaTS= OR jamaica sTS=) OR (serbiaTS= OR serbiaTS=) OR (botswanaTS= OR botswanaTS= OR botswana sTS=) OR (jordanTS= OR jordanTS=) OR SouthAfricaTS= OR (brazilTS= OR brazilTS= OR brazil sTS= OR brazilsTS=) OR (kazakhstanTS= OR kazakhstanTS= OR kazakhstan sTS=) OR (saint luciaTS= OR (saintTS= AND luciaTS=) OR saint luciaTS= OR (stTS= AND luciaTS=) OR st luciaTS=) OR (bulgariaTS= OR bulgariaTS=) OR (lebanonTS= OR lebanonTS= OR lebanon sTS=) OR (vincent, st[Investigator] OR st vincent[Author] OR st vincent[Investigator]) OR (chinaTS= OR chinaTS= OR china sTS= OR chinasTS=) OR (libyaTS= OR libyaTS=) OR (surinameTS= OR surinameTS= OR surinamTS=) OR (colombiaTS= OR colombiaTS= OR colombia sTS=) OR (republic of north macedoniaTS= OR (republicTS= AND northTS= AND macedoniaTS=) OR republic of north macedoniaTS= OR macedoniaTS=) OR (thailandTS= OR thailandTS= OR thailand sTS=) OR (costa ricaTS= OR (costaTS= AND ricaTS=) OR costa ricaTS=) OR (malaysiaTS= OR malaysiaTS= OR malaysia sTS=) OR (turkeyTS= OR turkeyTS= OR turkey sTS= OR turkeysTS= OR turkeysTS=) OR (cubaTS= OR cubaTS=) OR (indian ocean islandsTS= OR (indianTS= AND oceanTS= AND islandsTS=) OR indian ocean islandsTS= OR maldivesTS= OR maldiveTS=) OR (turkmenistanTS= OR turkmenistanTS=) OR (dominicaTS= OR dominicaTS=) OR (micronesiaTS= OR micronesiaTS= OR (marshallTS= AND islandsTS=) OR marshall islandsTS=) OR (micronesiaTS= OR micronesiaTS= OR tuvaluTS=) OR (dominican republicTS= OR (dominicanTS= AND republicTS=) OR dominican republicTS=) OR (mauritiusTS= OR mauritiusTS=) OR (venezuelaTS= OR venezuelaTS= OR venezuela sTS=) OR (equatorial guineaTS= OR (equatorialTS= AND guineaTS=) OR equatorial guineaTS=) OR (mexicoTS= OR mexicoTS= OR mexico sTS= OR mexicosTS=) OR (africaTS= OR africaTS= OR africa sTS= OR africasTS=) OR (caribbean regionTS= OR (caribbeanTS= AND regionTS=) OR caribbean regionTS=) OR (central americaTS= OR (centralTS= AND americaTS=) OR central americaTS=) OR LatinAmericaTS= OR (south americaTS= OR (southTS= AND americaTS=) OR south americaTS=) OR (asiaTS= OR asiaTS=)	

19	#17 OR #18	2,783,855
20	#19 AND #16 AND #11 AND #6 AND #3	487

Supplemental 2 : Impacts of PM_1 on cardiovascular diseases

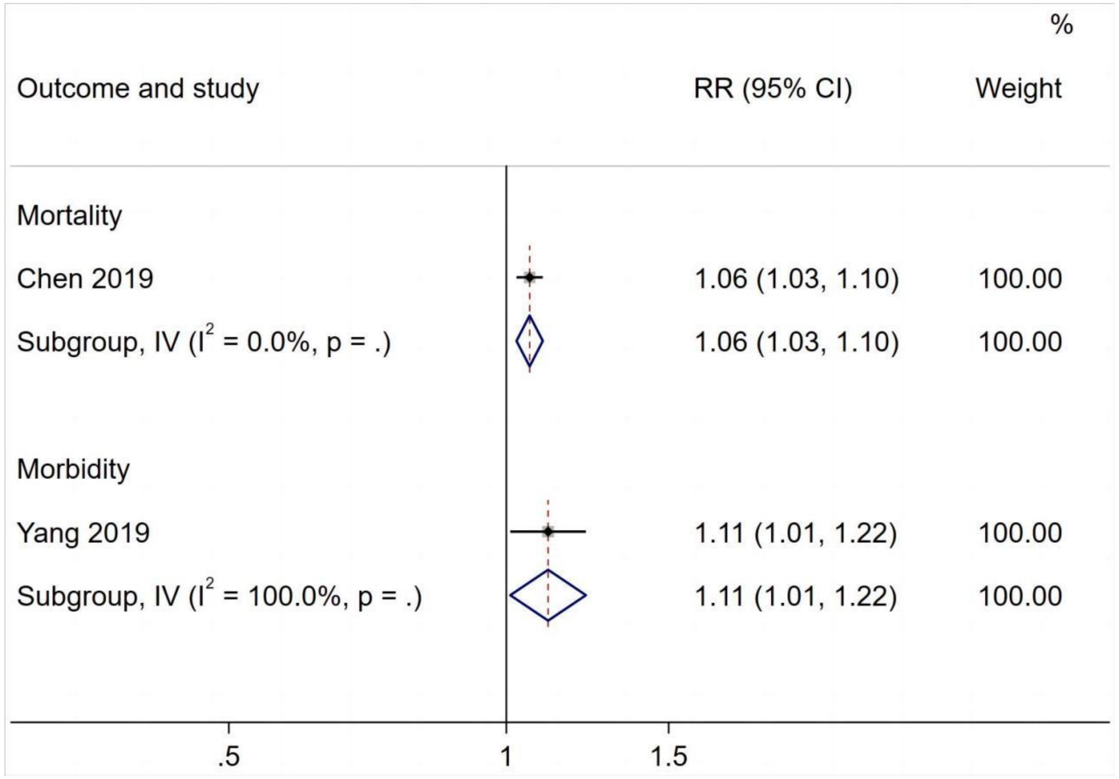

Supplement: Supplementary file 1 [file Data_Sheet_1.pdf]
